# mTORC1 pathway activity biases cell fate choice

**DOI:** 10.1038/s41598-024-71298-2

**Published:** 2024-09-06

**Authors:** Yuntao Wang, Monika Papayova, Eleanor Warren, Catherine J. Pears

**Affiliations:** Department of Biochemistry, University of Oxford, South Parks Road, Oxford, OX1 3QU UK

**Keywords:** *Dictyostelium discoideum*, mTORC1, Rapamycin, Cell fate choice, Set1, Cell biology, Developmental biology

## Abstract

Pluripotent stem cells can differentiate into distinct cell types but the intracellular pathways controlling cell fate choice are not well understood. The social amoeba *Dictyostelium discoideum* is a simplified system to study choice preference as proliferating amoebae enter a developmental cycle upon starvation and differentiate into two major cell types, stalk and spores, organised in a multicellular fruiting body. Factors such as acidic vesicle pH predispose amoebae to one fate. Here we show that the mechanistic target of rapamycin complex 1 (mTORC1) pathway has a role in cell fate bias in *Dictyostelium*. Inhibiting the mTORC1 pathway activity by disruption of Rheb (activator Ras homolog enriched in brain), or treatment with the mTORC1 inhibitor rapamycin prior to development, biases cells to a spore cell fate. Conversely activation of the pathway favours stalk cell differentiation. The Set1 histone methyltransferase, responsible for histone H3 lysine4 methylation, in *Dictyostelium* cells regulates transcription at the onset of development. Disruption of Set1 leads to high mTORC1 pathway activity and stalk cell predisposition. The ability of the mTORC1 pathway to regulate cell fate bias of cells undergoing differentiation offers a potential target to increase the efficiency of stem cell differentiation into a particular cell type.

## Introduction

Stem cells proliferate in culture and can be induced to differentiate into alternative cell types and so have potential in therapeutic medicine, for example to replace particular sets of neurons in neurodegenerative diseases. However, an inability to reliably control the fate of mammalian multi-potent stem cells with high efficiency currently limits this potential. Therefore an increased understanding of the mechanisms that determine the transition to differentiation and choice between alternative cells fates is required. The social amoeba *Dictyostelium discoideum* offers an excellent system to study this as proliferating cells can differentiate down two alternative pathways^1^. This eukaryotic organism proliferates as single cells that aggregate to form a multicellular organism upon starvation, driven by extracellular cAMP^2^. After 24 h, the final fruiting body contains two major cell types with a ball of spores supported above the surface by a stalk of dead, vacuolated cells. Differentiation of the amoebae into one or other cell type can also be driven in monolayer cultures allowing characterisation of differentiation in both monolayer and in a multicellular context^3^. Cell fate choice of the proliferating amoebae can be biased by a number of factors, revealing mechanisms of symmetry breaking in the seemingly uniform population^4^. This bias can be assessed by taking advantage of the unusual mechanism to generate a fruiting body by aggregation of cells. Mixing cells of different genetic backgrounds or with distinct growth histories at the onset of starvation generates chimeric fruiting bodies and the percentage of cells forming spores in the final structure can be compared with the input percentage^3^. Cell fate bias can also be demonstrated in monolayer cultures as cells with a stalk fate bias show increased sensitivity to low concentrations of the stalk inducing factor DIF-1 to induce expression of prestalk-specific markers, suggesting a mechanistic basis^3,5,6^. Examples of factors causing bias are cell cycle position at the onset of starvation^7^, acidic vesicle (lysosome) pH^8^ ATP levels^9^, glycogen reserves^10^ and calcium (Ca^2+^) content^11^.

Transient release of Ca^2+^ into the cytosol via gated channels signals a variety of cellular processes including differentiation. Channels include the inositol 1,4,5-trisphosphate receptor (IP_3_R) on neutral Ca^2+^ stores and Two Pore Channels (TPCs) which are located on acidic Ca^2+^ stores. In *Dictyostelium,* changes in cytosolic Ca^2+^ in response to early developmental signals such as extracellular cAMP or DIF-1 are not detectable in cells which lack the gene encoding the single protein with homology to the mammalian IP_3_R (*iplA*^*-*^ cells)^12^. High cellular Ca^2+^ content is associated with a stalk cell bias, though the molecular basis behind this is not known, including whether the different internal Ca^2+^ stores play distinct roles. *Dictyostelium discoideum* has a single gene encoding a homologue of a Two Pore Channel protein 2 (TPC2), and *tpc2*^*−*^ cells show reduced cytosolic Ca^2+^ levels relative to parental AX2 cells, and slightly delayed and lower rise in cytosolic Ca^2+^ in response to cAMP during early development^13^. These cells also showed an increase in pH of acidic vesicles, both features associated with a spore fate bias. Interestingly, *tpc2*^*−*^ cells also show increased phosphorylation of 4E-binding protein 1 (4E-BP1) during growth and early development. This phosphorylation is catalysed by the mechanistic target of rapamycin complex 1 (mTORC1)^13^. mTORC1 activity is regulated by amino acid availability^14,15^, and is a key player in mammalian cell fate choice, for example in T cells^16–18^, stem cell pluripotency^19,20^ and tissue regeneration^21,22^. AMP-activated protein kinase (AMPK) is an important energy sensor and metabolic regulator that antagonizes mTORC1^23,24^, whereas the monomeric guanine nucleotide binding protein Rheb is a direct activator of mTORC1^25^. The mTOR pathway is conserved in *Dictyostelium* and plays a role in both growth and development^26,27^, and dysregulated mTOR pathway activity via genetic modulation of regulators alters cell proliferation and developmental processes^28–31^.

Here we report that, despite the lower cytosolic Ca^2+^ and higher vesicle pH of acidic vesicles that have been associated with a prespore cell fate bias, *tpc2*^*−*^ cells show a stalk cell fate bias as assessed in both chimaeric structures formed with parental AX2 cells, and by increased sensitivity to induction of expression of a stalk cell-specific reporter in monolayer culture in response to DIF-1. This raised the possibility that altered mTORC1 activity might underly the cell fate preference. Disruption of genes encoding regulators to increase (*ampk*^*−*^) or reduce (*rheb*^*−*^) mTORC1 activity demonstrate that high mTORC1 pathway activity lead to a stalk fate preference. mTORC1 inhibition by rapamycin prior to development is sufficient to cause a spore fate bias, demonstrating a role at the onset of development. The histone H3K4 methyltransferase Set1 plays a role in regulation of gene expression at the growth development transition in *Dictyostelium*^32^ and here we show that *set1*^*−*^ cells show a stalk cell bias and increased mTORC1 pathway activity. Taken together, this data the level of mTORC1 activity provides a mechanistic basis for cell fate choice in *Dictyostelium* that may be more widely applicable.

## Results

### *tpc2*^*−*^ cells show a stalk cell fate bias

**Fig. 1:**
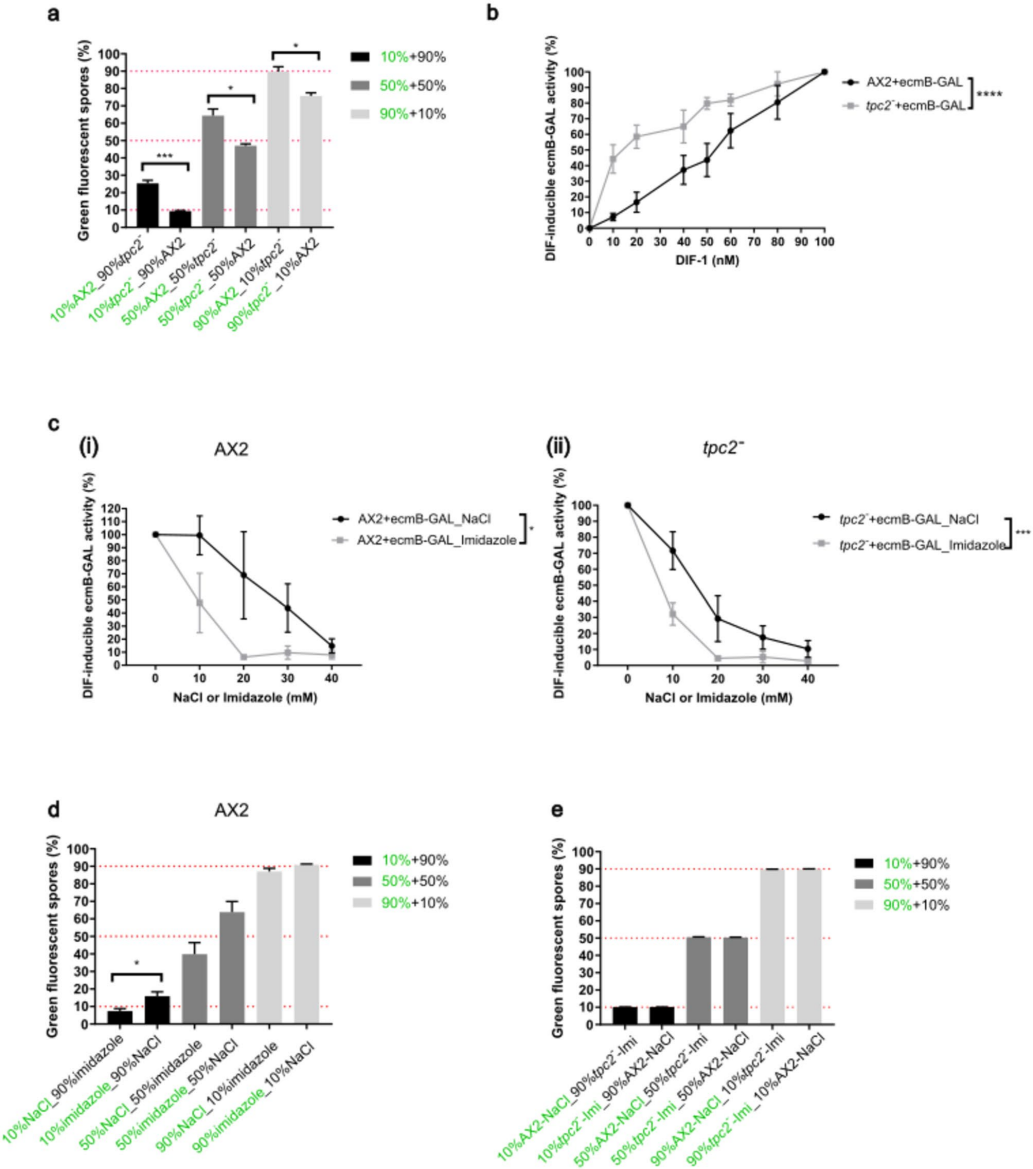
Cell fate bias in *tpc2*^*−*^ cells. (**a**) Chimeric development. Exponentially growing AX2 and *tpc2*^*−*^ cells were resuspended in KK2 and then aliquots of both strains labelled with 10 μM CellTracker™ Green CMFDA Dye. AX2 and *tpc2*^*−*^ cells were mixed in 10/90, 50/50 or 90/10 ratios prior to development on nitrocellulose filters, with labelled and unlabelled proportions indicated in green or black font. Spores were harvested and fixed on coverslips mounted with DAPI-containing VECTASHIELD for imaging by fluorescent microscopy. The number of spores labelled by cell tracker and the total number of spores were quantified using ImageJ software. Mean (N = 3) ± S.E.M. Student *t-*test (two-tailed) was performed for each mixing ratio: *: P < 0.05; ***: P < 0.001. (**b**) Monolayer stalk marker induction assays. Exponentially growing AX2 and *tpc2*^*−*^ cells expressing the DIF-1-inducible stalk cell marker *ecmB*-GAL were resuspended in stalk buffer with 5 mM cAMP and 50 μM cerulenin for 24 h followed by *ecmB*-GAL induction by a range of DIF-1 concentrations for 24 h. β-galactosidase enzymatic activity was quantified by endpoint absorbance measurement at 575 nm. The expression levels were normalized to the 100 nM DIF-1 treated groups defined as 100%. Mean (N = 3) ± S.E.M. Two-way ANOVA: ****: P < 0.0001. This result was verified with two clones: TPC22B20 (N = 3) and TPC1E14 (N = 1, data not shown). (**c**) Monolayer stalk induction assays were performed using (i) AX2 and (ii) *tpc2*^*−*^ cells expressing *ecmB-*GAL in the presence of increasing concentrations of imidazole or NaCl (0–40 mM) throughout development and the *ecmB*-GAL expression induced by 100 nM DIF-1. Activity was normalized relative to the non-treated groups (0 mM NaCl or 0 mM imidazole) defined as 100%. Mean (N = 3) ± S.E.M. (**d**) Chimeric development of AX2 cells following 18-h pre-treatment with 20 mM imidazole or NaCl (osmotic control) or (**e**) 4-h pre-treatment of AX2 cells with 20 mM NaCl and *tpc2*^*−*^ cells with 20 mM imidazole was performed as in (**a**). Mean (N = 3) ± S.E.M.

The bias to form spore or stalk cells in the final fruiting body is influenced by both vesicle pH and cellular Ca^2+^ content, both of which are altered in *tpc2*^*−*^ cells^13^, predicting a prespore bias. To investigate this, spore formation was assessed by generating chimeric fruiting bodies in which different percentages of *tpc2*^*−*^ cells were mixed with parental AX2 cells at the onset of starvation. Fluorescent labelling of one cell type allowed the percentage of spores derived from that cell type in the final fruiting bodies to be quantified in comparison to the input ratio. Stalk cell preference was assessed by determining the sensitivity of the cells to low concentrations of DIF-1 to induce expression of a DIF-inducible gene, *ecmB*-GAL, in monolayer assays. In both assays *tpc2*^*−*^ cells showed a stalk cell fate tendency as this strain contributed a lower percentage of spores than input in the final fruiting body and showed increased *ecmB*-GAL expression at lower concentrations of DIF-1 (Fig. 1a,b). This was unexpected as the reduced cytosolic Ca^2+^ levels and higher vesicle pH in *tpc2*^*−*^ cells have both been associated with a spore fate bias. Therefore, in this case neither of these parameters was defining the bias.

Treatment with weak bases such as imidazole, which neutralises the pH of acidic compartments, predispose cells to a spore fate^33^. In monolayer assays, imidazole treatment throughout cell differentiation is able to decrease the sensitivity of *tpc2*^*−*^ cells to DIF-1 induction of *ecmB*-GAL expression, as in parental AX2 cells (Fig. 1c). The use of pharmacological reagents to alter bias in mixing experiments is challenging, as all cells in chimeric structures are exposed to any added reagent during development. Therefore, we first determined whether pre-treatment of cells with imidazole only during growth, prior to the onset of development, would be sufficient to demonstrate a cell fate bias. Imidazole treatment of AX2 cells for 18 h during growth led to a spore cell bias when mixed with control cells, demonstrating that altering the vesicle pH prior to the onset of development is sufficient to induce cell fate bias (Fig. 1d). When *tpc2*^*−*^ cells pre-treated with imidazole during growth were developed in chimaeras with parental control cells, the *tpc2*^*−*^ cells no longer showed a stalk cell tendency (Fig. 1e). Therefore, treatment with weak bases is still able to alter the bias of *tpc2*^*−*^ cells.

### mTORC1 activity in populations with a cell fate bias

**Fig. 2:**
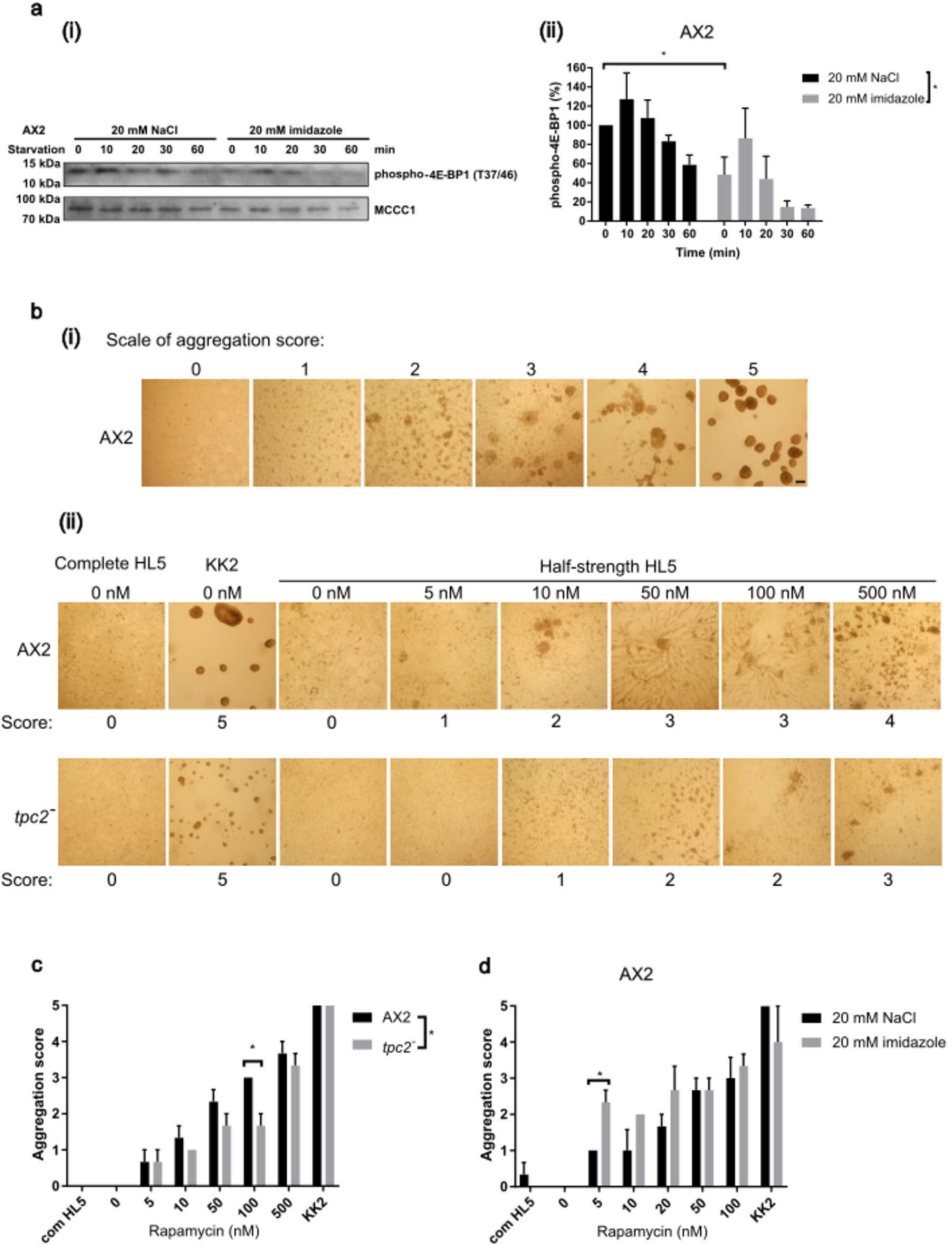
mTORC1 pathway activity upon starvation correlates with cell fate tendency. (**a**) Phosphorylation levels of 4E-BP1. Exponentially growing AX2 cells treated during growth with 20 mM imidazole or NaCl were starved by resuspending in HKC buffer for the times shown. The resulting western blots were probed with anti-phospho-4E-BP1 (T37/46) (upper panel). Methylcrotonyl-CoA carboxylase (MCCC1) loading control (Davidson et al., 2013) was visualised using Streptavidin. The PVDF membrane was cut into two parts for probing for the two proteins of different molecular weight. (i) Cropped sections of one representative blot are shown, N = 3. (ii): The intensities of bands were quantified by Image Studio Lite Software (Ver 5.2) and normalized to 0 min time point of the control group (NaCl treated) defined as 100%. Mean (N = 3) ± S.E.M. Two-way ANOVA: P < 0.05. Student *t*-tests were used to compare 0 min (non-starved) time points between two groups: *: P < 0.05. (**b**) Rapamycin-induced aggregation. Log-phase cells were plated and incubated with either full-strength/complete HL5 or half-strength HL5, in the presence of various concentrations of rapamycin or vehicle control DMSO (0 nM). A positive control for aggregation was included, using KK2 with DMSO. After 22-h, the aggregates in each well were imaged. (i) Standards for scoring system used to represent different aggregation levels. Scale bar 100 μm. (ii) A representative example of rapamycin-induced aggregation assay in half-strength HL5 medium with AX2 or *tpc2*^*−*^ cells in the presence of various concentrations of rapamycin. (**c, d**) Aggregation of AX2 and *tpc2*^*−*^ cells (**c**) and in AX2 cells pre-treated with 20 mM imidazole or NaCl for 4 h (**d**) was scored at a range of rapamycin concentrations as described in (b). Mean (N = 3) ± S.E.M. Two-way ANOVA: *: P < 0.05. Student *t*-test (two-tailed) for each treatment group.

Factors known to influence cell fate such as growth in high glucose, ATP levels and acidic vesicle pH are also well characterized to modulate the activity of mTORC1^34^. Phosphorylation of the mTORC1 substrate 4E-BP1 decreases rapidly upon starvation in *Dictyostelium*^27^ and *tpc2*^*−*^ cells show an increased and prolonged phosphorylation of 4E-BP1 during growth and upon starvation^13^. Interestingly, AX2 cells grown in the presence of 20 mM imidazole prior to the onset of development showed reduced levels of phosphorylation of 4E-BP1 compared to the control cells measured by western blot using a phospho-specific antibody, although this still decreased upon starvation (Fig. 2a). This reduction in 4E-BP1 phosphorylation levels correlated with spore fate tendency whereas the increased level seen in *tpc2*^*−*^ cells correlates with a stalk cell bias.

4E-BP1 is the only characterised substrate of mTORC1 in *Dictyostelium* and no robust alternative to monitor mTORC1 pathway activity has been identified. However, inhibition of mTORC1 by rapamycin treatment of cells in half-strength growth medium is sufficient to trigger aggregate formation^27^, mimicking some of the events of early development. Therefore, the sensitivity of cells to induction of aggregation by low concentrations of rapamycin could be alternative measure of mTORC1 pathway activity as cells with higher mTORC1 activity would require higher levels of rapamycin to induce aggregation. To test this, AX2 and *tpc2*^*−*^ cells were plated in half-strength HL5 growth medium, in the presence of increasing concentrations of rapamycin and the degree of aggregation scored after 22 h (Fig. 2b,c). For ease of comparison we used a scoring system to rapidly assess the degree of aggregation in different conditions. In AX2 cells rapamycin induced aggregation in a dose dependent manner. *tpc2*^*−*^ cells required higher concentrations of rapamycin to induce aggregation, consistent with the increased 4E-BP1 phosphorylation and higher mTORC1 pathway activity. This assay was then used to confirm the decreased level of mTORC1 pathway activity following imidazole pre-treatment prior to the onset of starvation as the imidazole treated AX2 cells showed greater aggregation at lower rapamycin concentrations, with 5 nM rapamycin causing significant aggregation in the imidazole treated cells which required 50 nM in control cells (Fig. 2d).

### Genetic manipulation of mTORC1 activity

**Fig. 3:**
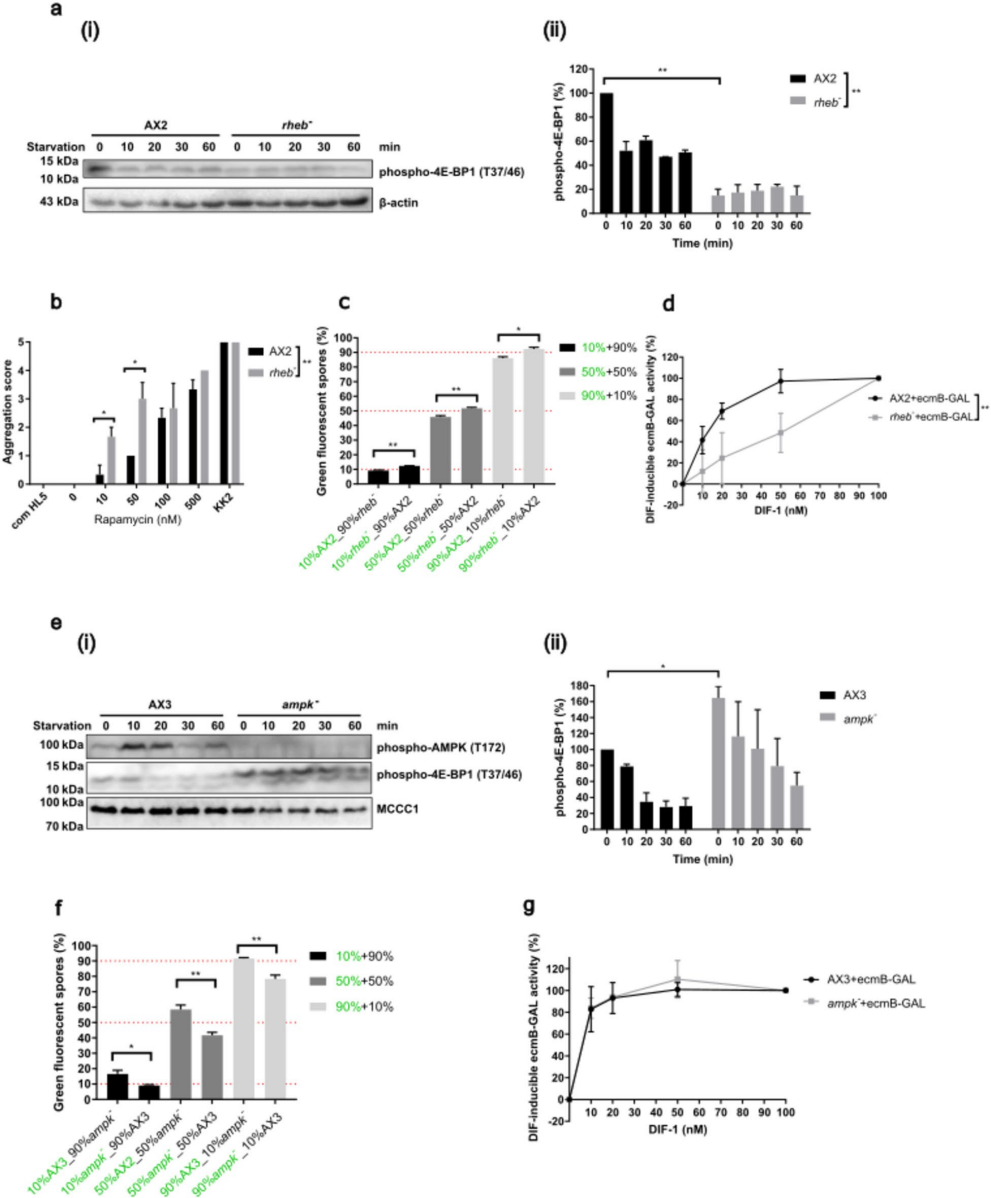
Genetic manipulation of mTORC1 pathway activity affects cell fate bias. (**a**) (i) One representative western blot (cropped) (N = 2) of phospho-4E-BP1 in AX2 and *rheb*^*−*^ cells starved for one hour. β-actin was used as loading control. The membrane was cut into strips for probing for proteins of different molecular weight. (ii) Quantification of phosphorylation levels of 4E-BP1 in AX2 and *rheb*^*−*^, normalized to 0 min starvation of AX2 group defined as 100%. Mean (N = 2) ± S.E.M. (**b**) AX2 and *rheb*^*−*^ strains were assessed in rapamycin-induced aggregation assays. Mean (N = 3) ± S.E.M. (**c**) Labelled spore percentages following chimeric development of AX2 and *rheb*^*−*^ cells in different ratios. Mean (N = 3) ± S.E.M. This result was verified with two independent clones: *rheb*^*−*^_I_12_1 (N = 3) and *rheb*^*−*^_I_3_1 (N = 2, data not shown). (**d**) β-Gal activity following monolayer stalk induction assays of AX2 and *rheb*^*−*^ cells expressing *ecmB*-GAL. Mean (N = 4) ± S.E.M. (**e**) (i) Western blot analysis of cells harvested at the times shown following starvation of AX3 and *ampk*^*−*^ cells using phospho-AMPK (T172) and phospho-4E-BP1 (T37/46) antibodies. MCCC1: Loading control. Cropped panels of one representative blot shown, N = 3. The membrane was cut into strips for probing for proteins of different molecular weights. (ii) Quantification of phospho-4E-BP1 levels in western blots. AX3 0 min starvation (in growth) was defined as 100%. Mean (N = 3) ± S.E.M. (**f**) Labelled spore percentages following chimeric development of AX3 and *ampk*^*−*^ cells mixed at different ratios. Mean (N = 3) ± S.E.M. (**g**) β-Gal activity following monolayer stalk induction assays of parental AX3 and *ampk*^*−*^ cells expressing *ecmB*-GAL.

To test whether altering mTORC1 pathway activity is sufficient to cause a cell fate predisposition, strains with mutations in regulatory pathways affecting mTORC1 activity were tested. To decrease mTORC1 activity using a genetic approach, cells were generated in which the single gene encoding an orthologue of Rheb in *Dictyostelium* was disrupted. Development of the *rheb*^*−*^ cells was indistinguishable from parental AX2 cells. (Figure S1). The level of phospho-4E-BP1 was reduced in growing *rheb*^*−*^ cells and the level did not decrease upon starvation, although any decrease could be below the detection limits of this approach (Fig. 3a). Consistent with a reduction in mTORC1 pathway activity, these cells showed an increased aggregate formation in half-strength HL5, at low concentrations of rapamycin (Fig. 3b). This decreased pathway activity predicted a spore fate bias and indeed *rheb*^*−*^ cells gave rise to an increased proportion of spores when developed as chimaeras with parental AX2 cells (Fig. 3c), and a decreased sensitivity to DIF-1 induction of *ecmB*-GAL expression in monolayers (Fig. 3d).

*Dictyostelium ampk*^*−*^ cells with a disrupted gene encoding the catalytic α subunit have an extended doubling time, prolonged cell proliferation in growth and can form developmental structures^30^. AMPK is activated by phosphorylation, so phospho-specific antibodies against these sites can be used to directly measure its level of activity. Consistent with previous reports, parental AX3 cells show an increase in levels of phospho-AMPK within 10 min of starvation as well as a decrease in phospho-4E-BP1 levels over a similar time course (Fig. 3e). *ampk*^*−*^ cells show no detectable levels of phospho-AMPK but show an increased basal level of phospho-4E-BP1 although this did still decrease upon starvation, so other pathways are also regulating mTORC1 at this stage (Fig. 3e). In our hands rapamycin was unable to cause aggregate formation of *ampk*^*−*^ cells in half-strength HL5 (data not shown). The reason for this is unclear, but the phospho-4E-BP1 data is consistent with an increase in mTORC1 pathway activity and led to a prediction that these cells would show a stalk cell fate bias. This was apparent in mixing experiments (Fig. 3f), although no difference was seen in sensitivity to DIF-1 in monolayer assays (Fig. 3g).

### Influence of mTORC1 activity at different developmental stages

**Fig. 4:**
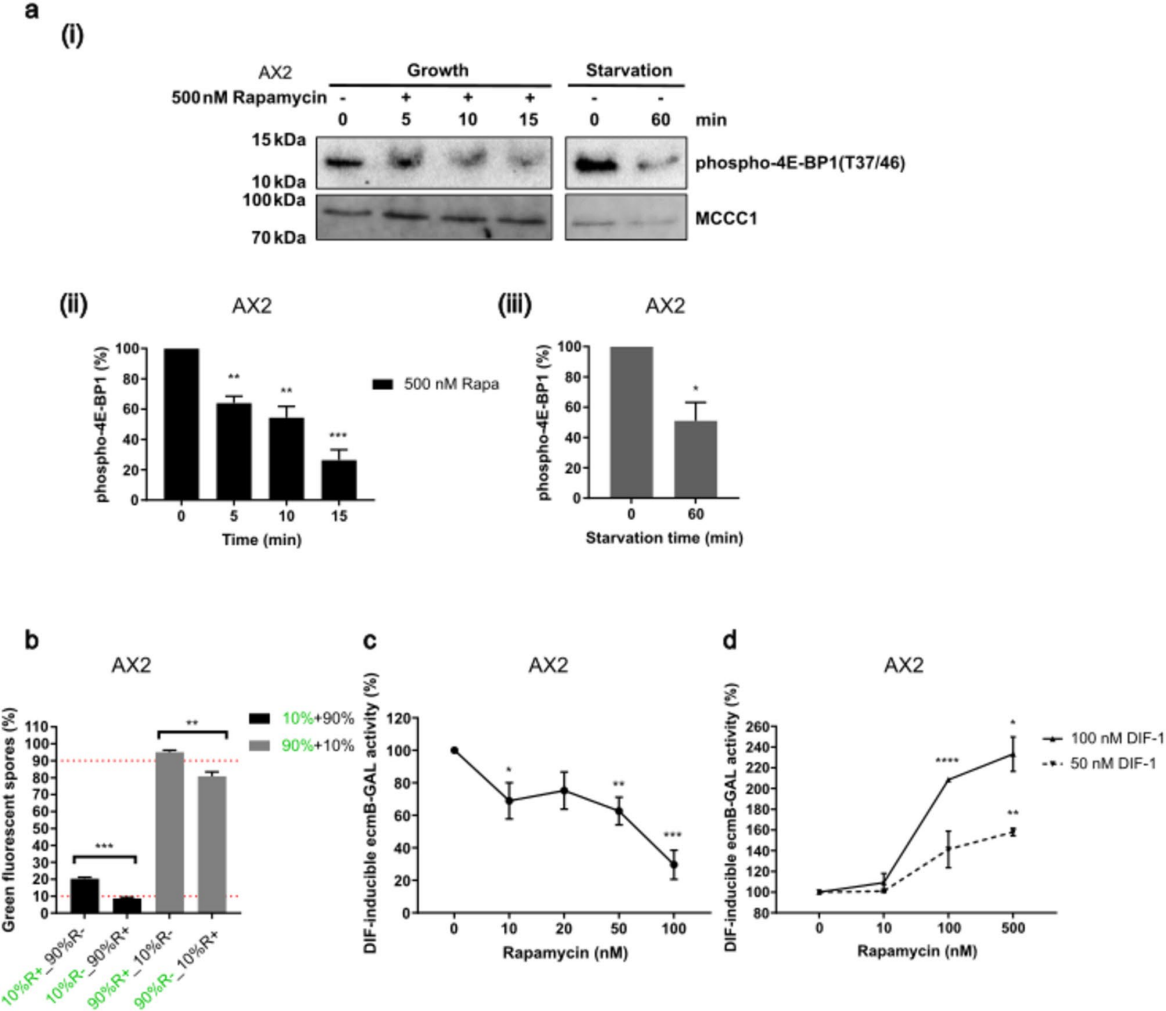
Pharmacological inhibition of mTORC1 pathway activity at different stages causes different cell fate bias. (**a**) (i) Log-phase AX2 cells were resuspended in HL5 growth medium with or without 500 nM rapamycin. Phosphorylation of 4E-BP1 was detected by western blot. A control for reduction of phospho-4E-BP1 levels following one-hour starvation was included. One representative blot (cropped) from each is shown, N = 3. The membrane was cut into strips for probing for proteins of different molecular weight. Quantification of (ii) the phospho-4E-BP1 levels from rapamycin treatment in growth or (iii) control experiments of 60 min starvation, normalized to the untreated groups (0 min). Mean (N = 3) ± S.E.M. (**b**) Exponentially growing cells were treated with 100 nM rapamycin (R +) or DMSO vehicle control (R-) for 4 h in HL5 growth medium before chimeric development. Mean (N = 3) ± S.E.M. (**c**) *ecmB*-GAL expression induced by 100 nM DIF-1 in AX2 cells pre-treated during growth for 4 h with increasing concentrations of rapamycin following monolayer assays. The expression levels were normalized to the untreated (0 nM) group. Mean (N = 4) ± S.E.M. (**d**) Monolayer stalk induction assays were performed with the indicated concentrations of rapamycin present throughout the assay with either 50 or 100 nM DIF-1. Mean (N = 2) ± S.E.M.

To confirm the role of mTORC1 activity in cell fate predisposition, a pharmacological approach was used. Rapamycin is a well-studied mTORC1 inhibitor which has been widely used in *Dictyostelium*^27,35^, whereas no other mTORC1 specific inhibitors are known to be effective in this organism. Growing cells were incubated with 500 nM rapamycin, for up to 15 min and the amount of phosphorylated 4E-BP1 decreased within 5 min, showing reduced activation levels of the mTORC1 pathway. Given the short time scales, this is consistent with direct inhibition of mTORC1 activity (Fig. 4a). Pre-treatment of cells with rapamycin for 4 h during growth led to an increased number of spores derived from rapamycin pre-treated cells when mixed with untreated cells (Fig. 4b). This spore fate bias was confirmed using the monolayer stalk induction assay as incubating cells during growth with increasing concentrations of rapamycin led to lower levels of *ecmB*-GAL induction, consistent with reduced activity of the mTORC1 pathway activity at the onset of development leading to a spore fate bias (Fig. 4c).

Interestingly, if cells were not pre-treated with rapamycin but instead rapamycin was added throughout monolayer development, an increase in *ecmB*-GAL expression was seen in response to low concentrations of DIF-1 (Fig. 4d), suggestive of a stalk bias which is the opposite of that seen in cells that have been treated during growth. This suggests that mTORC1 pathway activity has different effects on cell fate bias at different times of development: low activity at the growth development transition favouring a spore fate while low activity later in development favouring a stalk cell fate. This complexity could explain the unchanged sensitivity of *ampk*^*−*^ cells to DIF-1 in monolayer assays.

### Gene expression regulated by mTORC1 pathway activity

**Fig. 5:**
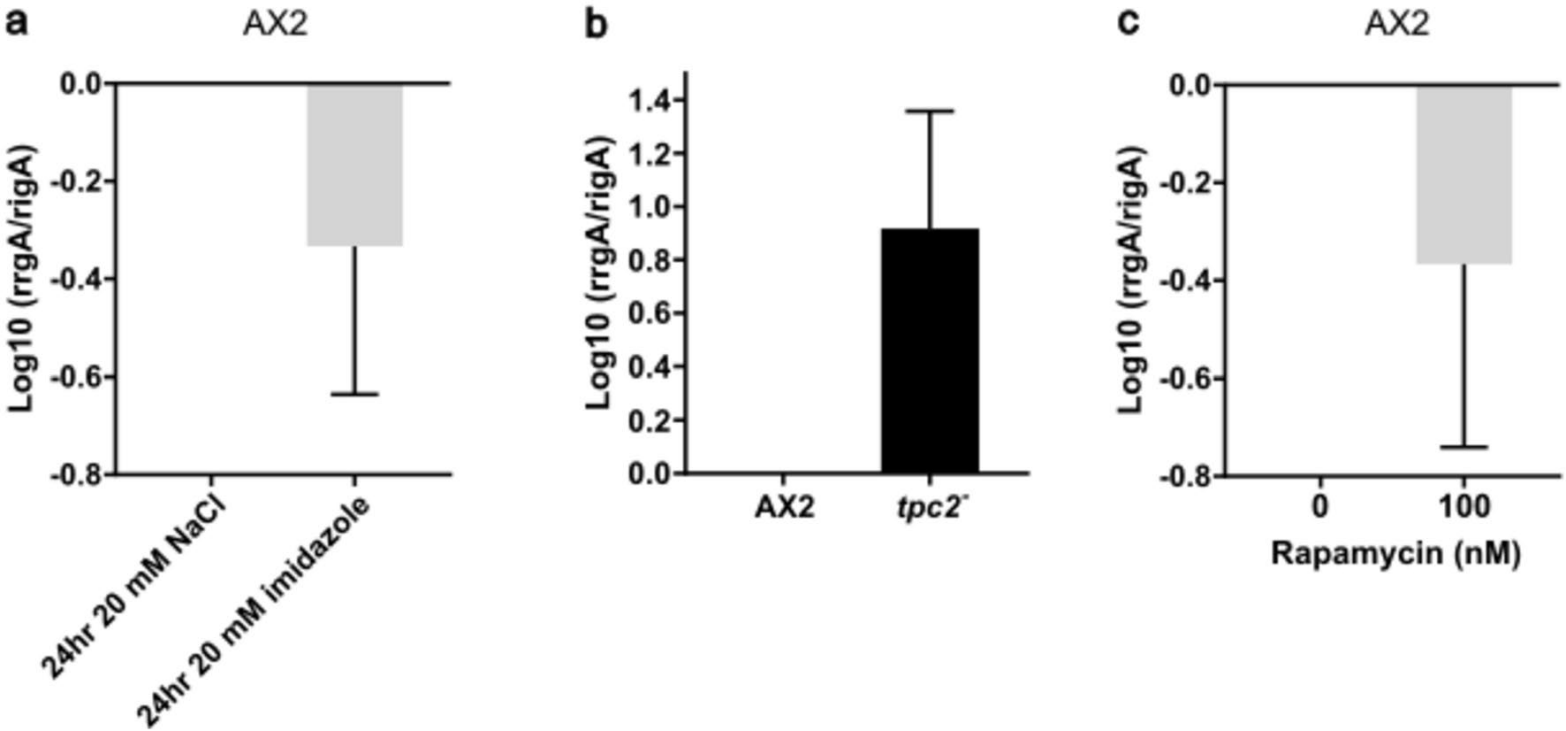
The ratio of expression of *rrgA/rigA* during growth. Exponentially growing AX2 cells were harvested and lysed in TRIzol for total RNA extraction, following by cDNA synthesis. The expression levels of *rrgA* and *rigA* were determined by qPCR, comparative ΔΔCt analyses were conducted^60^. *rrgA/rigA* expression ratios in (**a**) AX2 cells incubated in HL5 with 20 mM NaCl (osmotic control) or 20 mM imidazole for 24 h (N = 3), (**b**) AX2 (parental control) and *tpc2*^*−*^ cells (N = 3) as well as (**c**) AX2 cells with 100 nM rapamycin pre-treatment or vehicle control DMSO (0 nM) for 24 h. All data sets were normalized to relevant control groups, where control cell lines or untreated DMSO groups were calibrator controls set to have an *rrgA/rigA* ratio of 1 and the ratios for experimental groups or lines expressed as a fraction of this. A negative log10 value then represents a lower ratio. Mean (N = 3) ± S.E.M. Student *t-*test (two-tailed): *: P < 0.05.

Analysis of RNAseq data from growing cells with spore or stalk cell fate bias has identified that the ratio of expression of two genes, *rrgA* (DDB_G0268600) and *rigA* (DDB_G0274655), in growing cells prior to their entry into development is a biomarker of cell fate bias, including of cells grown in more acidic media^36^. A low ratio of expression of these two genes predicts a spore fate bias and a high ratio a stalk cell bias. We have shown that treatment with the weak base imidazole during growth also leads to a spore cell fate bias. Therefore, we used quantitative RT-PCR to determine the ratio of expression following pre-treatment with imidazole during growth and a low ratio of expression was seen, predictive of the spore fate bias seen (Fig. 5a). Conversely, stalk cell-biased *tpc2*^*−*^ cells showed a high expression ratio during growth (Fig. 5b). To determine if mTORC1 inhibition was sufficient to cause this change in ratio, cells were treated during growth with rapamycin (Fig. 5c) and indeed the ratio decreased in line with the reduced sensitivity to DIF-1 induction of *ecmB*-GAL expression in monolayers and with increased spore numbers in chimaeras. This is consistent with the level of mTORC1 activity in growing cells contributing towards setting a pattern of gene expression which, in turn, predisposes cell fate.

### mTORC1 pathway activity in growing cells is regulated by histone H3 lysine 4 methylation by Set1

**Fig. 6:**
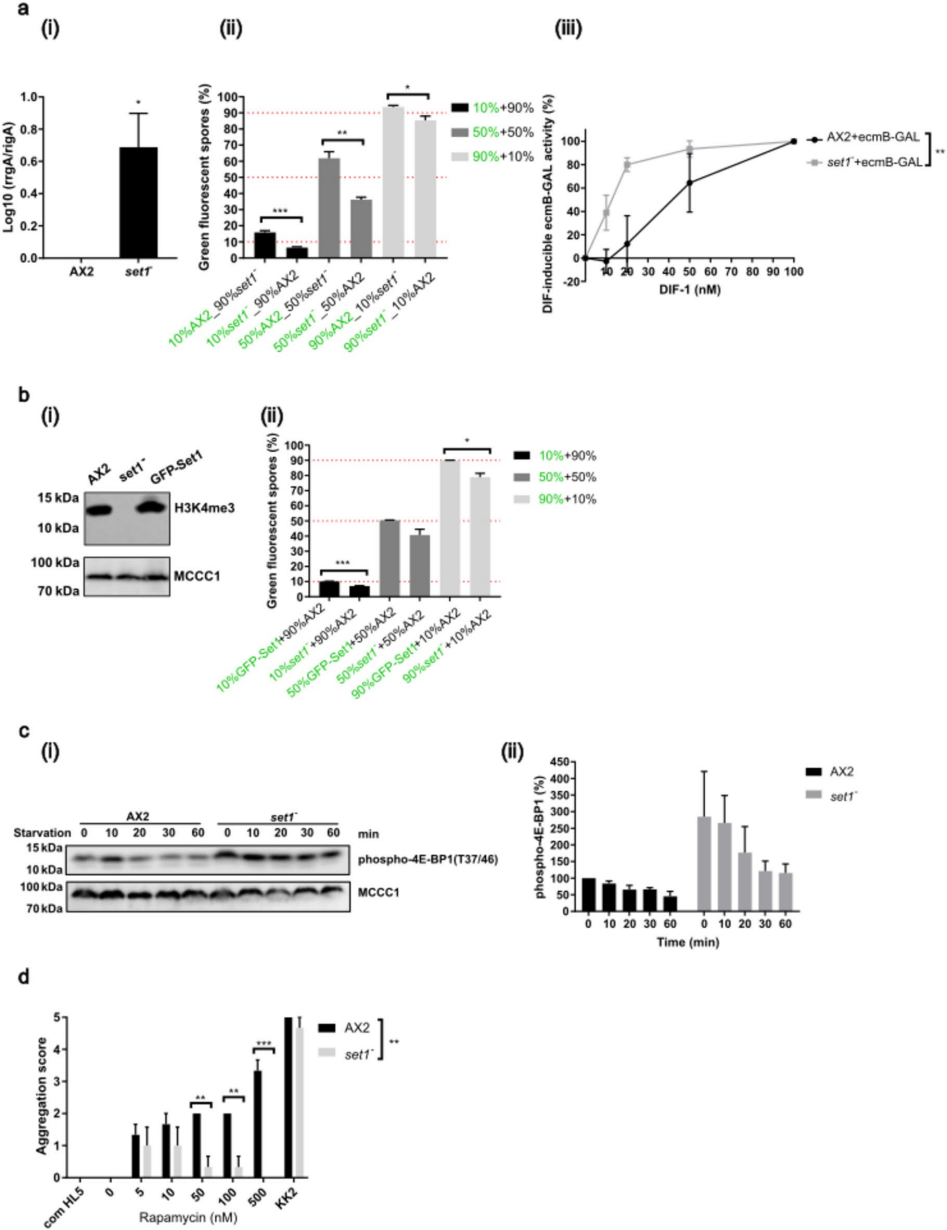
Cell fate bias is dependent on histone H3K4 methylation. (**a**) (i) *rrgA/rigA* expression ratio in *set1*^*−*^ cells compared with AX2 following quantitative RT-PCR. Mean (N = 3) ± S.E.M. (ii) Labelled spore percentages following chimeric development of AX2 and *set1*^*−*^ cells mixed in different ratios. Mean (N = 4) ± S.E.M. (iii) β-gal activity was determined following monolayer stalk induction assays of AX2 and *set1*^*−*^ cells expressing *ecmB*-GAL treated with 0–100 nM DIF-1. Mean (N = 4) ± S.E.M. (**b**) (i) Whole cell lysates of exponentially growing AX2, *set1*^*−*^ and *set1*^*−*^ cells expressing GFP-Set1 were analysed by western blot using antibodies specific for H3K4me3. MCCC1: loading control. Cropped panel of one representative blot shown, N = 3. The membrane was cut into parts for probing for proteins of different molecular weight. (ii) Chimeric development of unlabelled AX2 cells mixed with labelled *set1*^*−*^ or GFP-Set1 cells and the percentage of labelled spores shown. Mean (N = 3) ± S.E.M. (**c**) Phospho-4E-BP1 levels on starvation were determined in AX2 and (i) *set1*^*−*^ (N = 4) and (ii) quantification of phosphorylation levels were normalized to 0 min starvation of AX2 cells (in growth) defined as 100%. Cropped panels from one representative experiment shown. The membrane was cut into parts for probing for proteins of different molecular weight. (**d**) Rapamycin induced aggregation of *set1*^*−*^ cells compared to AX2 cells was assessed in half-strength HL5 medium. Mean (N = 3) ± S.E.M.

An important factor in control of gene expression at the growth/development transition in *Dictyostelium* is methylation of lysine 4 on histone H3 by the Set1 methyltransferase^32^. *set1*^*−*^ cells show early expression of a number of developmentally regulated genes and more rapid aggregation. To determine if this pathway plays a role in gene expression leading to cell fate predisposition, the ratio of expression of *rrgA/rigA* was determined in *set1*^*−*^ growing cells and found to be higher than in parental AX2, predictive of a stalk cell fate bias. In mixing experiments with parental AX2 cells *set1*^*−*^ cells were under-represented in the spore population and in monolayer, *set1*^*−*^ cells showed increased *ecmB*-GAL expression at low concentrations of DIF-1, both assays demonstrating bias towards a stalk cell fate (Fig. 6a). Both trimethylation of histone H3 on K4 and the stalk cell bias were reversed by expression of GFP-Set1 in *set1*^*−*^ cells (Fig. 6b).

Both Set1 activity and mTORC1 activity can lead to a cell fate bias. To determine if the two were linked, and if so, whether Set1 regulated mTORC1 pathway activity or vice versa, the mTORC1 pathway activity levels at the onset of development were tested in *set1*^*−*^ cells. *Set1*^*−*^ cells showed an increased level of phospho-4E-BP1 during growth compared to parental AX2 cells (Fig. 6c). Consistent with this, *set1*^*−*^ cells also showed a reduced sensitivity to low rapamycin concentrations in half-strength HL5 (Fig. 6d). At high concentrations of rapamycin, *set1*^*−*^ cells failed to form aggregates at all, though the reason for this is not known. Both of these assays reveal an increased mTORC1 pathway activity. Therefore, strains deficient in H3K4me show increased mTORC1 activity during growth. This data is consistent with Set1-dependent gene expression leading to an alteration in mTORC1 activity which, in turn, regulates the gene expression pattern in growing cells that determines cell fate tendency.

## Discussion

An in depth understanding of the pathways involved in cell fate decisions in culture and in the context of multicellular development will be fundamental to the potential use of stem cell therapy. *Dictyostelium* provides an excellent system to study this as it has a relatively simple multicellular development lifestyle and bias between the two major cell fates can be studied in both multicellular and monolayer context. A number of factors have been demonstrated to cause cell fate bias, but the molecular basis behind these has remained elusive. The biases reported here and by others^3^, lead to relatively modest changes in the percentage of spores in fruiting bodies but these are reflected in changes to DIF sensitivity in the monolayer situation. Therefore an understanding of even relatively small biases could lead to an increased ability to drive cells down a preferred pathway in monolayers. Strains which show a spore cell bias in chimaeras can also show an increased number of spores when developed in isolation^37^. Stalk bias could be explained in terms of sensitivity to the stalk inducing molecule DIF-1. As DIF-1 levels rise in the mound, cells with an early prestalk bias, randomly scattered in the aggregate, are more sensitive to low levels of DIF-1 and therefore start to differentiate first down this pathway^3^. They then act as DIF-1 sinks, reducing the level of DIF-1 and therefore supressing stalk cell differentiation of surrounding cells^4^. Altered speed of movement and expression of cell adhesion proteins to stabilise interaction with cells of the same fate, lead to cell sorting by the tipped aggregate stage of development. However, cells that are unable to synthesise DIF-1 are only deficient in one subtype of prestalk cell (pstO cells) but otherwise make fruiting bodies with spore and stalk cells^6^, so this cannot be the full explanation. However this does highlight differences in cells during growth and at the onset of development as an important determinant of sensitivity to factors such as DIF-1 and therefore a cell fate bias.

Two assays were in this study used to monitor cell fate bias—one by determining the number spores in chimeric multicellular structures to assay bias in a multicellular context and also a more defined monolayer assay. The promoter used to drive marker expression in this latter assay, *ecmB* is directly induced by DIF-1. It is expressed in a subset of stalk cells late in development but is used here as a marker for DIF-1 sensitivity to changes in gene expression. The two assays agree except in the case o*f ampk*^−^ cells where a bias was apparent in multicellular but not monolayer assays. This is consistent with the known spore fate bias of *ampk*^*−*^ cells^30^. The reason for the difference in monolayer culture is not known but may reflect a different role for AMPK late in development distinct from a bias during growth. The opposing roles for the AMPK/mTORC1 pathways at different developmental stages in the monolayer assay is also suggested by the reversal of sensitivity to *ecmB-*GAL induction by DIF-1 when rapamycin is present throughout monolayer development as opposed to prior to development at the growth/development transition. AMPK regulates many other pathways in the cell, not only mTORC1 activity, and the gene disruption will affect these pathways throughout development, not just at the transition to development, making these results in monolayer development hard to interpret. In future, analysis of DIF-1 sensitivity of *ampk*^*−*^ cells treated with rapamycin, or with agents to directly inhibit or activate AMPK, either during growth or at different stages during monolayer development could help unravel this complexity. Rheb has a much more specific role in regulation of mTORC1 and the phenotype of *rheb*^*−*^ cells and the mixing experiments with *ampk*^*−*^ cells are consistent with the importance of mTORC1 activity in cell fate bias. The importance of the timing of pathway activation during growth prior to the onset of development is demonstrated by the differential effect on cell fate bias of treating cells with the rapamycin either during growth (spore fate bias) or during development (stalk cell bias). A role for reciprocal activities of AMPK and mTORC1 in late development has been previously hypothesized^38^. AMPK has been suggested to play a role in DIF-1-regulated phosphorylation of several proteins including the calcineurin catalytic subunit during development^39^.

*tpc2*^*−*^ cells have at least two features which are known to predispose cells to a spore fate: slightly reduced cytosolic Ca^2+^ responses and reduced acidity of acidic vesicles^13^. Here we show that, despite this, these cells show a stalk cell fate bias in both monolayer assays and development in chimaeras, in agreement with others^40^ and this bias still responded to neutralisation of acidic vesicles by weak bases. As many of the factors causing bias are predicted to regulate mTORC1 activity (glycogen reserves, ATP levels)^9^, we then investigated whether the prolonged phosphorylation of 4E-BP1 in these cells might play a role in the bias. Treatment of cells with imidazole to specifically neutralise acidic vesicles^33^ reduced mTORC1 activity and caused a bias towards spore fate. Importantly, pre-treatment of cells with imidazole or rapamycin during growth was sufficient to cause cell fate bias, revealing that mTORC1 activity during growth and/or early development was likely to be important. mTOR pathway activity also alters T cell fate decisions, for example, rapamycin-induced mTORC1 inhibition leads to anergic status of CD4+ T cells and memory CD8+ T cell differentiation, while disruption of the *mtor* gene in T cells leads to loss of differentiation of distinct T helper cell lineages (such as T_H_1, 2 and 17)^41^. AMPK is also essential for T_H_1 and T_H_17 accumulation but not differentiation capability of naïve CD4+ T cells^42^. An antagonistic role for mTORC1 and AMPK also exists in regulation of mouse embryonic stem cell pluripotency during pharmacologically induced autophagy^43^ and in response to energetic stress^44^.

**Fig. 7:**
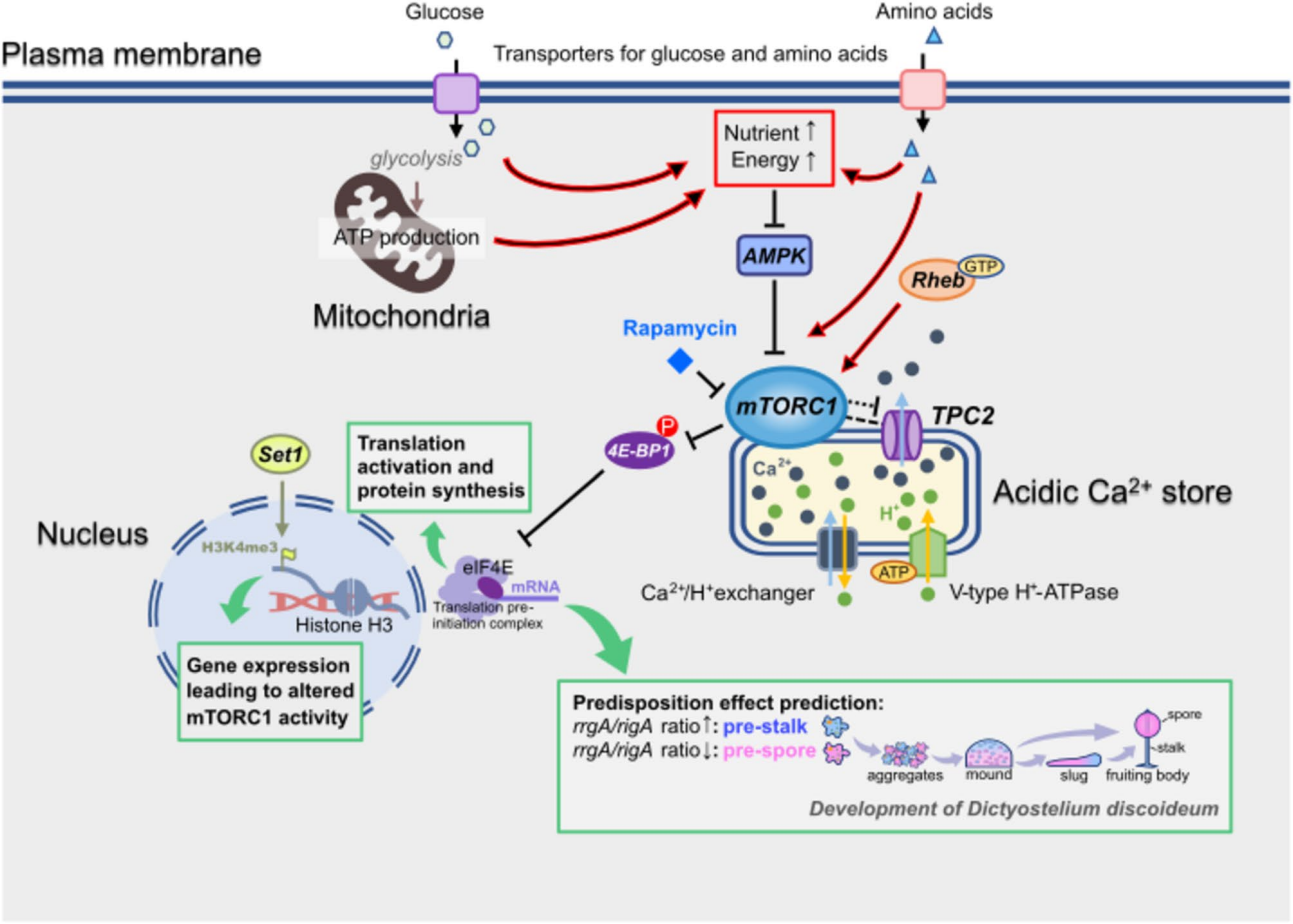
Model for the role of mTORC1 in cell fate bias in *Dictyostelium*. *Dictyostelium* mTORC1 activity can be modulated, directly or indirectly, by Rheb, AMPK, amino acid availability and Set1-dependent gene expression. The mTORC1 activity levels at the onset of development leads to cell fate bias, potentially though regulation of translation.

The activation level of the mTORC1 pathway is a marker of cell fate bias and manipulating activity either genetically by disruption of regulatory pathway components, or pharmacologically by treatment with rapamycin, is sufficient to cause bias (Fig. 7 for summary). Deletion of the gene encoding Rheb^35,45^ and the reciprocal relationship between mTORC1 and AMPK activity are established in *Dictyostelium*^27,46^. Two methods were used where possible to monitor mTORC1 pathway activity, phosphorylation of the established mTORC1 substrate 4E-BP1 and sensitivity of the cells to low concentrations of rapamycin to form aggregates in half-strength medium. In mammalian cells 4E-BP1 and the ribosomal S6 kinase (S6K) are two well-studied downstream effectors of mTORC1 and their phosphorylation levels are used as readouts of in vivo mTORC1 activity in eukaryotes^47^ where both regulate cap-dependent translation initiation^48,49^. There is no obvious orthologue of this S6K protein encoded in the *Dictyostelium* genome to use as a second marker of pathway activity (discussed in^50^). Like others, we have not identified an antibody against total 4E-BP1 that reliably recognises the *Dictyostelium* protein, so the changes detected here using phospho-specific antibodies could be due to changes in levels of the protein as well as changes in phosphorylation state^27,28,46^. The short time scales for reducing levels of phosphorylated protein detected at the onset of starvation and on rapamycin treatment are consistent with dephosphorylation and correlate with rapamycin sensitivity to induce aggregation. However, even if the differences in phosphorylation level are due to alterations in protein amount, especially between different strains, they still act as a marker for the overall activity of the pathway if phosphorylated 4E-BP1 is a marker for cell fate bias. mTORC1 regulates protein synthesis through the amount of phosphorylated of 4E-BP1, so an important consequence of altered mTORC1 activity could be a shift in the level of translation which in turn could lead to differential changes in levels of some proteins. Examining the proteome of growing cells with different mTORC1 pathway activity and cell fate bias could reveal changes with potential to underlie the mechanism of bias. It would be of interest to determine whether the bias seen in multicellular development on alteration of mTORC1 activity also leads to a change in the number of spores when cells are developed in isolation^37^.

Post-translational histone modifications are well-conserved with roles in regulating gene expression^51^. The histone methyl transferase Set1 plays an important role in the transcriptional changes at the onset of *Dictyostelium* development. *Set1*^*−*^ cells show dysregulation of expression of genes in genomic clusters and importantly premature activation of genes involved in the development programme during growth^32^. The H3K4me3 modification is enriched at gene promoters and works at least in part through recruitment of a histone acetyl transferase complex^52^. Here we show that *set1*-null cells have a stalk cell fate bias. The increase in 4E-BP1 phosphorylation and increase in the concentration of rapamycin required to induce any aggregation in *set1*^*−*^ cells shows that Set1 normally supresses mTORC1 activity perhaps through regulation of expression of mTORC1 regulatory pathway components. The ratio of expression of *rrgA/rigA* in growing cells was used as a readout for the cell fate bias at the level of transcription^36^, and gave results consistent with the mixing and monolayer assays. These genes were identified following RNAseq of cells with bias and act as a marker for a range of bias conditions. Other markers, such as expression of *omt12*^53^ or *rasD*^54^, act as markers for either prespore or prestalk cell fate bias but the ratiometric method described here was chosen as it monitors both cell types. The fact that Set1 regulates mTORC1 activity and inhibition of mTORC1 by rapamycin treatment alters the ratio of *rrgA/rigA* expression is consistent with a model where reciprocal regulation of expression of *rrgA* and *rigA*, and genes that regulate bias, is downstream of mTORC1 rather than dependent on Set1 directly (Fig. 7).

An understanding of the role for the mTORC1 complex activity in cell fate choice will allow increased control of these decisions in stem cell populations, both in cell culture and organisms. This would be facilitated by the increased availability of pharmacological reagents able to specifically manipulate mTORC1 activity in both bioengineered and natural systems for studying developmental biology.

## Materials and methods

### *Dictyostelium* cell culture

*Dictyostelium* axenic strain AX2 was used as a parental cell line for *tpc2*^*−*^^13^, s*et1*^*−*^^32^ and *rheb*^*-*^ cells, and AX3 is the parent of *ampk*^*−*^^46^. Both AX2 and AX3 cells were obtained from the *Dictyostelium* stock centre (dictybase.org). Cells were grown on SM agar plates in association with bacteria *Klebsiella aerogenes* (KA) or in shaking suspension in HL5 medium with 1% glucose (55.5 mM glucose) at 220 rpm, at 22 °C. Cells were harvested during exponential growth and by centrifugation at 1225×*g*, 3 min. Where possible key experiments were repeated on independent clones.

### Electroporation of *Dictyostelium*

Cells were electroporated as described in Gaudet, et al.^55^. Exponentially growing cells were washed with KK2 (2.59 g KH₂PO₄, 1.04 g K₂HPO₄ in 1L milliQ dH2O) twice and resuspended in chilled H50 (20 mM HEPES, 50 mM KCl, 10 mM NaCl, 1 mM MgSO₄·7H₂O, 5 mM NaHCO₃, 1 mM NaH₂PO₄·2H₂O, adjusted pH 7.0) to 5 × 10^7^ cells/mL. In each pre-chilled cuvette with 1 mm electrode gap (Bio-Rad, #1652083), a mixture of 95 μL cells mixed with 4–8 μg of plasmid DNA was added to a total volume of 100 μL. After 5-min incubation on ice, cells were exposed to two consecutive pulses of 650 V, 25 μF capacitance and 200 Ω resistance with 5-s recovery between pulses (GenePulser Xcell™, Bio-Rad). Immediately, electroporated cells were incubated on ice for 5 min, transferred to a 90 mm petri dish containing 10 mL fresh HL5 medium and kept at 22 °C. Antibiotic selection was applied the next day: 10 μg/mL G418 (Invitrogen), or 10 μg/mL blasticidin (Cambridge Bioscience).

### Chimeric development

The protocol for chimeric development (mixing experiment) was modified from published methods^3,56^. Exponentially growing cells were washed in KK2 twice and resuspended to 5 × 10^7^ cells/mL in KK2. Cells were split into two pools, left unlabelled (vehicle DMSO only) or stained with 10 μM CellTracker™ Green CMFDA Dye (5-chloromethylfluorescein diacetate, #C2925, Invitrogen) for 45 min at 22 °C and then washed with KK2 buffer for three times. Pools of labelled or unlabelled cells were resuspended in KK2 to the cell density of 5 × 10^7^ cells/mL and mixed in different proportions. 50 µL of the mixtures were plated onto nitrocellulose filters supported on buffered pads at 22 °C for development. All spores were harvested and resuspended in 50 µL KK2 buffer, and 30 µL spore suspension added to coverslips for 30 min. Coverslips were washed with 70% ethanol followed by 100% methanol. 2 μL VectaShield® Mounting Medium containing 1.5 µg/mL DAPI was then added. Spore visualization was performed using Olympus IX71 microscope under 100 × oil lens with exposure time of 120 ms for DAPI channel, 800 ms for FITC channel and 60 ms for DIC channel. Around 600 spores were counted for each.

### Monolayer stalk induction assay

This method was adapted from previous literature^3,57^. Early log phase cells containing a stalk reporter (*ecmB* promoter fused with lacZ^58^) for expressing β-galactosidase (*ecmB*-GAL) were harvested, washed with KK2 and resuspended at 2.32 × 10^5^ cells/mL in stalk medium (10 mM MES, 1 mM CaCl_2_, 20 mM KCl, 1 mM MgCl_2_, 20 mM NaCl, pH 6.2, with 100 μg/mL PenStrep) containing 5 mM cAMP and 50 µM cerulenin. 1.2 × 10^4^ cells in 50 μL were added into each well of a 96-well microplate, incubating for 24 h at 22 °C. The cells were then washed with KK2 twice. To induce stalk marker expression, 50 μL stalk medium was added lacking cAMP but with various concentrations of DIF-1 (from 0 to 100 nM) for individual wells for 24-h incubation at 22 °C. Lysis buffer (200 mM HEPES (pH 8.0), 2 mM MgSO_4_, 4% Triton X-100) with 2 mM Chlorophenolred-β-D-galactopyranoside (CPRG) (Roche) was applied to the cells. The 96-well plate then was incubated at 37 °C for 2 h. β-galactosidase activity was monitored by measuring optical density at 575 nm in microplate reader (BioTek 800TS), untreated (0 nM DIF-1) values were subtracted and values normalized to 100 nM DIF-1 treated groups defined as 100%.

### Rapamycin-induced aggregation assay

The method was adapted from previous literature^27^. Exponentially growing cells were collected and washed with KK2 twice. The cells were resuspended in KK2 buffer (positive control) or half-strength HL5 medium (normal HL5 diluted dH_2_O) to a density of 1.4 × 10^6^ cells/mL and 1 mL of cell suspension was plated in each well of a 12-well plate with the final cell density 4 × 10^5^ cells/cm^2^. A range of rapamycin concentrations or vehicle control DMSO were applied to the cells which were incubated at 22 °C for 22 h and imaged for aggregation assessment.

### Quantitative real-time RT-PCR (qPCR)

For qPCR analysis, total RNA was isolated from *Dictyostelium* cells using the TRIzol® Reagent (Invitrogen, #15596026) following the manufacturer’s protocol. 10 μg total RNA was purified to remove residual gDNA with TURBO DNA-free™ Kit (Ambion, #AM1907). 2 μg DNase treated total RNA was reverse transcribed to cDNA using SuperScript™ IV FirstStrand Synthesis System (Invitrogen, #18091050). Fast SYBRTM Green Master mix (Applied Biosystems, #4385612) was used following manufacturer’s protocol, and each 20-µL reaction containing 20 ng cDNA template and 200 nM of each forward and reverse primers in a well of a MicroAmp™ Fast Optical 96-Well Reaction Plate (Applied Biosystems, #4346907) was performed in StepOnePlus™ Real-Time PCR instrument (Applied Biosystems, #4376600). (95 °C, 20 s for initiation; 95 °C, 3 s; 60 °C, 30 s, for 40 cycles). The endogenous control *ig7* gene (*rnlA*, DDB_G0294034) was used for normalization^59^ (*ig7* primer sequences: *ig7*-F: 5′-tcgatcagagacgcaagtcg; *ig7*-R: 5′-caccccaacccttggaaact.). For the predisposition biomarker analyses, *rrgA* and *rigA* expression levels were assessed by qPCR (*rrgA*-F: 5′-gtagaatcaactgcatggaacaag; *rrgA*-R: 5′-gttaatatcgtaaacagtagtccaaac; *rigA*-F: 5′-atccagagtgtccatcagtag; *rigA*-R: 5′-ccgaaatggtgaccatggtg). Comparative ΔΔCt method was used for qPCR analysis^60^.

### *Dictyostelium rheb* gene disruption by CRISPR-Cas9

For *rheb* gene disruption, an all-in-one CRISPR/Cas9 vector pTM1285 vector^61^ was used. For designing sgRNA, Crispor (http://crispor.tefor.net/) was used for PAM sequence identification and prediction of sgRNA target specificity.

Two pairs of annealed guide RNA oligonucleotides were designed from the target gene sequence of *rheb* (YW55-F: 5′-agcacatagaaagatctgtgtaat; YW56-R: 5′-aaacattacacagatctttctatg; YW57-F: 5′-agcaaacaggtctgagtatggtag; YW58-R: 5′-aaacctaccatactcagacctgtt). 10 μM individual pairs of these guide RNA oligonucleotides were phosphorylated in 1 × T4 ligation buffer (containing 1 mM ATP) (NEB, #B0202S) and 5 units of T4 polynucleotide kinase (NEB, # M0201S) and annealed to each other by heating to 95 °C for 5 min, following by cooling to 25 °C at 5 °C/min. These were then inserted into the Bpi1 site of the pTM1285 entry vector obtained from NBRP Nenkin stock centre (nenkin.nbrp.jp) Method adapted from Sekine, et al.^61^.

2–3 μg of vectors were co-transfected into AX2 parental cells. Single colonies of candidate clones were selected and verified by PCR and sequencing of the *rheb* gene. Three independent clones with indels towards the N terminus of the protein were identified (Supplementary Fig. 1) and key experiments carried out on more than one independent clone.

### Western blotting

Exponentially growing cells were washed with HK buffer (10 mM HEPES, 10 mM KCl, adjusted pH 7.0) twice. The cells were resuspended in HKC development buffer (HK plus 250 μM CaCl_2_) to a density of 1.4 × 10^7^ cells/mL. At desired time points, 2 × 10^6^ cells were collected for whole cell lysates. The resulting PVDF membrane was cut into two parts for probing for proteins of different molecular weight (including a loading control), and each part (Amersham Hybond, #10600101) was blocked with 5% milk-TBST for one hour and then subjected to primary antibodies (phospho-4E-BP1 (Thr37/46, rabbit): 1:1000, Cell Signaling Technology, #2855; FLAG (rabbit): 1:2000, Sigma-Aldrich, #F7425; β-actin (mouse): 1:10,000, Santa-cruz, #sc-47778); H3K4me3 (rabbit): 1:2000^62^; or Alexa 680-conjugated streptavidin for MCCC1 loading control (1:1000, Invitrogen, #S21378)^63^ in 5% milk-TBST 4 °C overnight. Secondary antibodies were polyclonal goat anti-rabbit Immunoglobulins/HRP (1:10,000, Dako) or polyclonal goat anti-mouse Immunoglobulins/HRP (1:10,000, Dako). SuperSignal™ West Femto Maximum Sensitivity Substrate (Thermo Scientific™, #34095) was applied and all blots imaged for 2–10 min using Odyssey Fc imaging system (LI-COR, Lincoln, NE, USA). Image Studio Lite Ver 5.2 (LI-COR) was used for quantification, and intensities normalized to loading controls. Then, the values of treated/starved samples in various time points were normalized to the values of untreated/non-starved samples of the control groups (0 min) identified as 100%.

### Statistics

For quantification of data, the sample means $\overline{X }$ and standard error mean (S.E.M) were used and the number of independent experiments was indicated as N. Sample mean differences were compared using two-tailed student *t*-tests or two-way ANOVA with Sidak’s multiple comparisons analysed by Prism7 GraphPad and Microsoft Excel. P values and asterisks system define significance level (α = 0.05) and the asterisks showing significance are displayed on relevant figures: *: P < 0.05; **: P < 0.01; ***: P < 0.001; ****: P < 0.0001.

## Supplementary Information

Supplementary Information.

## Data Availability

Data is provided within the manuscript or supplementary information files.
