# Supplementary material for: mTORC1 pathway activity biases cell fate choice
Source: Sci Rep. 2024 Sep 6;14:20832. doi: 10.1038/s41598-024-71298-2 (PMC11379915; doi:10.1038/s41598-024-71298-2)
Supplement: Supplementary file 1 — Supplementary Information. [file 41598_2024_71298_MOESM1_ESM.pdf]

mTORC1 pathway activity biases cell fate choice

Yuntao Wang, Monika Papayova, Eleanor Warren and Catherine J. Pears

Figure S1. Verification of *rheb*$^{-}$ strain constructed by CRISPR/Cas9 method. (a) Design of CRISPR/Cas9 genetic disrupted *rheb*$^{-}$ strain. (i) Schematic map of *rheb* gene in chromosome 2 and primer positions. Two pairs of single guide RNA (sgRNA) (YW55+56 and YW57+58) were cloned separately into pTM1285 CRISPR/Cas9 all-in-one vector, both targeting exon 1 for genetic disruption after co-transfected into AX2 cells. YW43 and YW44 were sequencing primers for confirming the frameshift mutations. (ii) The sequencing results from PCR products of gDNA from *rheb*$^{-}$ mutants aligned to wildtype Rheb sequence. **(b)** Amino acid sequence alignment of *rheb*$^{-}$ mutant and wildtype Rheb proteins. Premature stop codons are highlighted in red. Clustal Omega and BioEdit software were used to generate alignment results for (A) and (B). **(c)** Nitrocellulose membrane development of AX2 and *rheb*$^{-}$ CRISPR/Cas9 strain (two clones: I_12_1 and I_3_1). Exponentially growing cells were harvested and washed in KK2 twice. Cells then were resuspended to the density of **5** $\times 10^{7}$ cells/mL and 10 µL of cells were plated onto the nitrocellulose membrane and three layers of Whatman filter paper soaked in 200 µL KK2 in each well of 12-well plates. Developmental structures from AX2 and two clones of *rheb*$^{-}$ were observed at 24 hrs. Scale bar: 1 mm.

Supplementary figure

Figure S1

a (i)

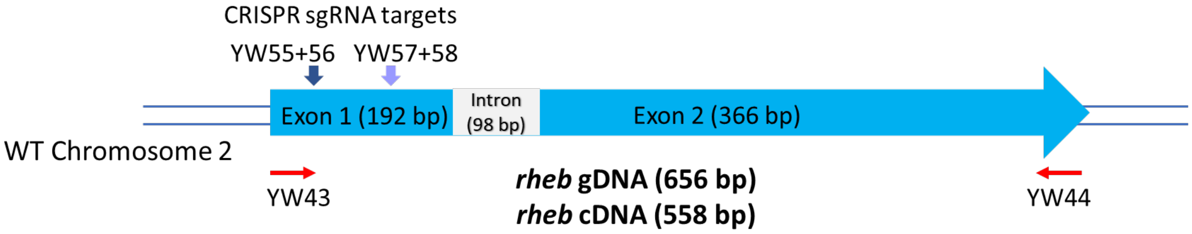

(ii)

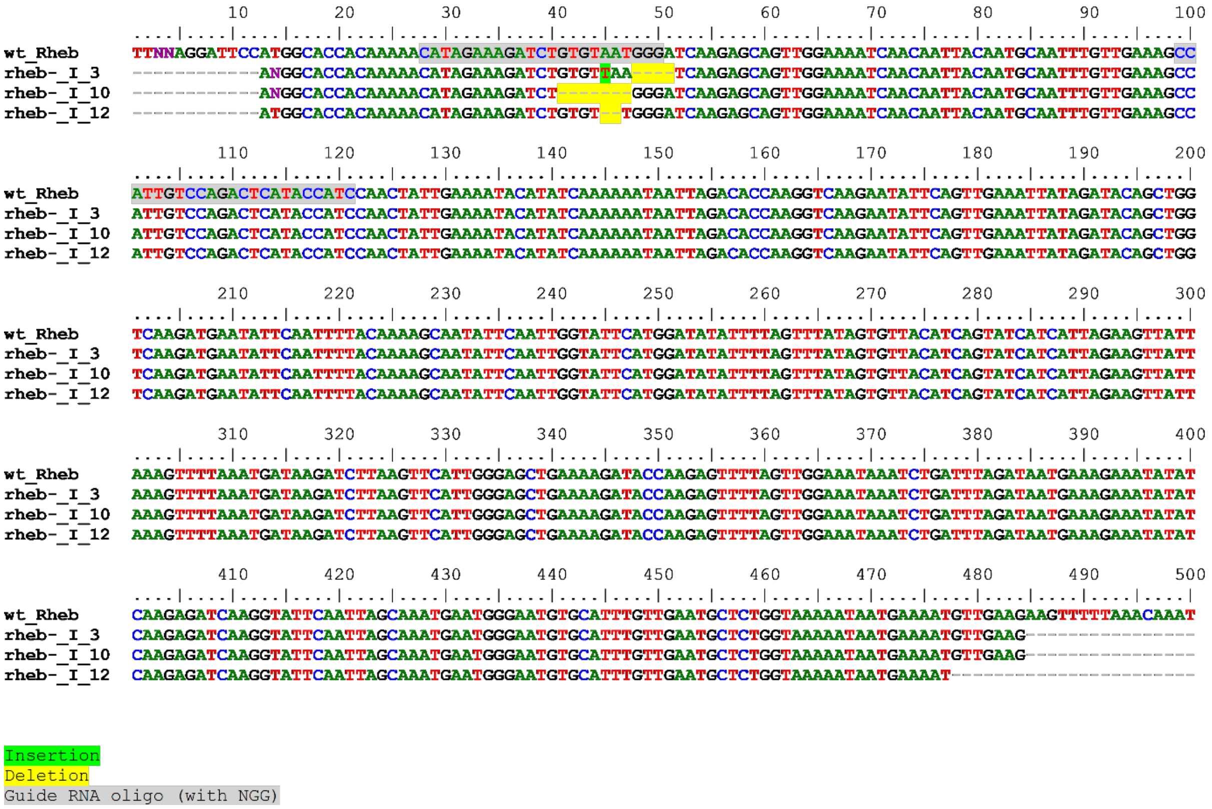

(b)

```
wt_Rheb_protein         MAPQKHRKICVMGSRAVGKSTITMQFVESHCPDSYHPTIENTYQKIIRHQGQEY----SV  56
rheb-_I_12_protein      MAPQKHRKICVGIKSSWKI-N----NYNAIC*KPLSRLIPSNY*KYISKNN*TP----RS  48
rheb-_I_10_protein      MAPQKHRKIW-DQEQLENQ-QLQCNLLKAIVQTHTIQLLKIHIKK*LDTKVKNIQLKL*I  56
rheb-_I_3_protein       MAPQKHRKICVNQEQLENQ-QLQCNLLKAIVQTHTIQLLKIHIKK*LDTKVKNIQLKL*I  57
                        *********    .             ::         :     * :  :

wt_Rheb_protein         EIIDTAGQDEYSILQKQYSIGIHGY-IL------VYSVTSVSSLEVIKVLNDKILSSLGA  109
rheb-_I_12_protein      RIFS*NYRYSWSR*IFNFTKAIFNWYSWIYFSL*CYISIIIRSY*SFK**DLKFIGS*KD  101
rheb-_I_10_protein      QLVKMNIQFYKSNIQL------------VFMD--IF*FIVLHQYHH*KLLKF*MIRS---  96
rheb-_I_3_protein       QLVKMNIQFYKSNIQL------------VFMD--IF*FIVLHQYHH*KLLKF*MIRS---  97
                        .:..   :  *                       :    : .    *  .  :: *

wt_Rheb_protein         EKIPRVLVGNKSDLDNERN---IS--RDQGIQLANEWECAFVECSGKNNENVEEVFKQIL  164
rheb-_I_12_protein      TKSFSWK*I*FR**KK-----YIKRSRYSISK*MG-------MCIC--*MLW-*---K--  134
rheb-_I_10_protein      --*VHWELKRYQEF*LEINLI*IMKEIYQEIKVFN-------*QMNGNVHLL-NALVKIM  142
rheb-_I_3_protein       --*VHWELKRYQEF*LEINLI*IMKEIYQEIKVFN-------*QMNGNVHLL-NALVKIM  143
                                              *     .  :  .                      :

wt_Rheb_protein         NEVNKGSTGPEPPQKEGCILM*  185
rheb-_I_12_protein      -**K------------------  135
rheb-_I_10_protein      KMLK------------------  146
rheb-_I_3_protein       KMLK------------------  147
                           :
```

(c)

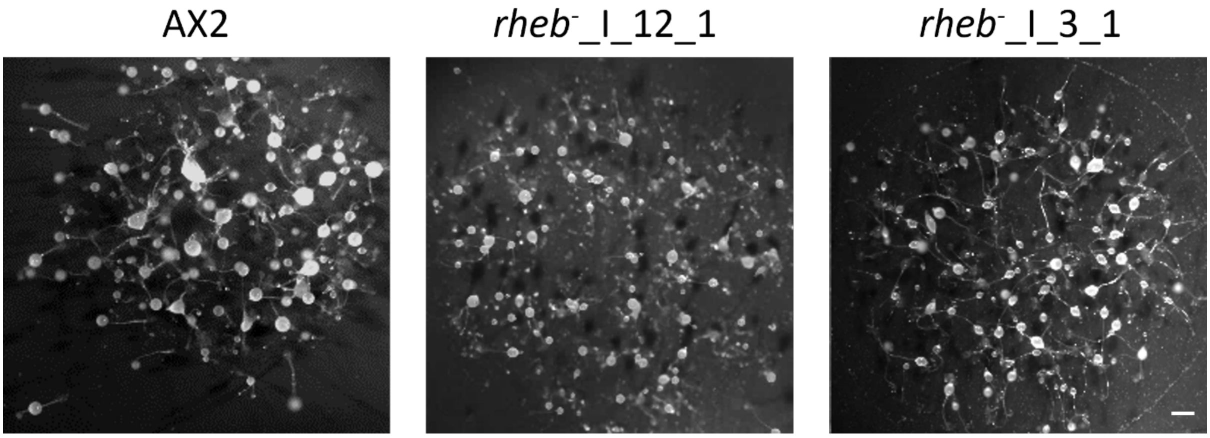

Original blots

Figure 2A (i)

Blot 1: Western blotting: phospho-4E-BP1 (T37/46) (rabbit, 1:1000)

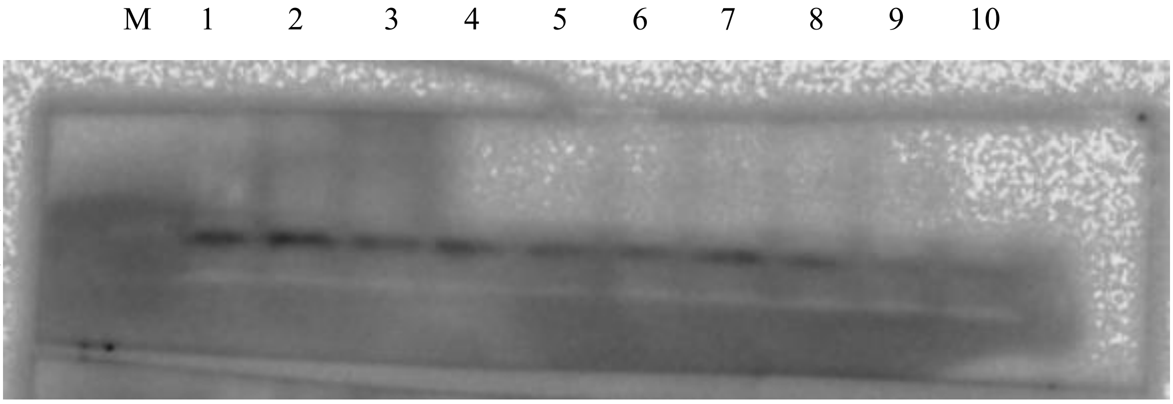

Blot 2: Western blotting: MCCC1 (Streptavidin, Alexa Fluo 680 conjugate, 1:1000)

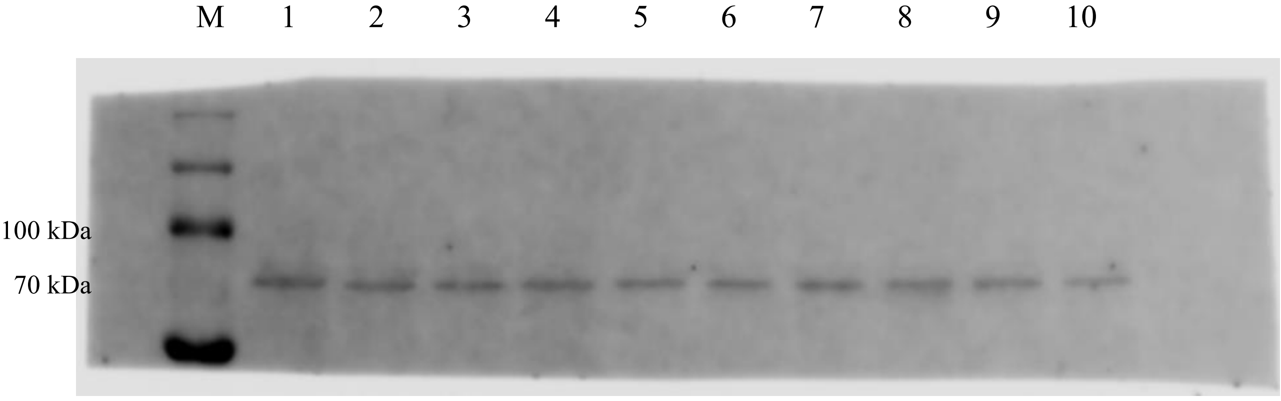

Lanes of Blot 1&2:

M: PageRuler Prestained Protein Ladder, 10 to 180 kDa (Thermo Scientific, #26619)

<u>**1-5 lanes:** whole cell lysates from AX2 cells pre-treated with 20 mM NaCl for 18 hrs during growth</u>

1: 0 min starvation (non-starved cells)

2: 10 min starvation

3: 20 min starvation

4: 30 min starvation

5: 60 min starvation

<u>**6-10 lanes:** whole cell lysates from AX2 cells pre-treated with 20 mM imidazole for 18 hrs during growth</u>

1: 0 min starvation (non-starved cells)

2: 10 min starvation

3: 20 min starvation

4: 30 min starvation

5: 60 min starvation

Figure 3A (i)

Blot 3: Western blotting: phospho-4E-BP1 (T37/46) (rabbit, 1:1000)

M 1 2 3 4 5 6 7 8 9 10

15 kDa
10 kDa

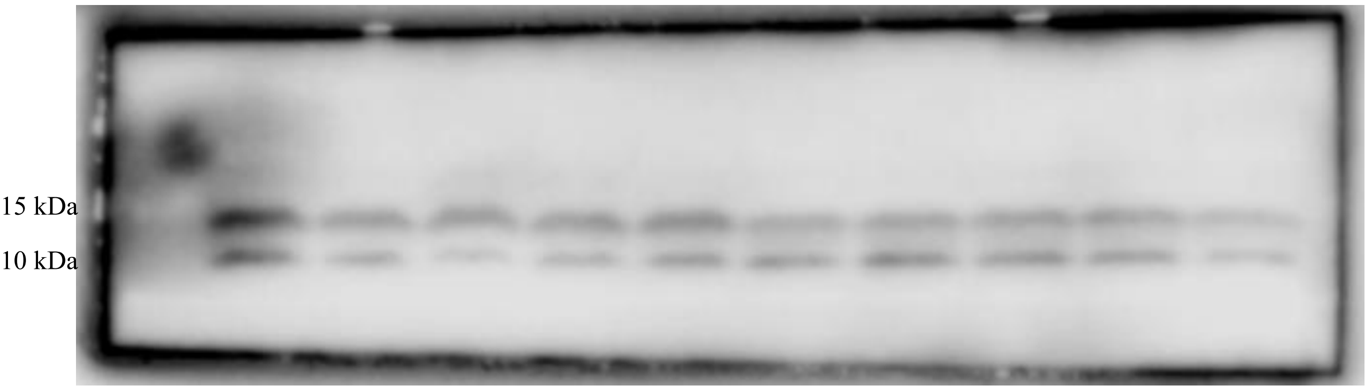

Blot 4: Western blotting: β-actin (mouse, 1:1000)

M 1 2 3 4 5 6 7 8 9 10

43 kDa

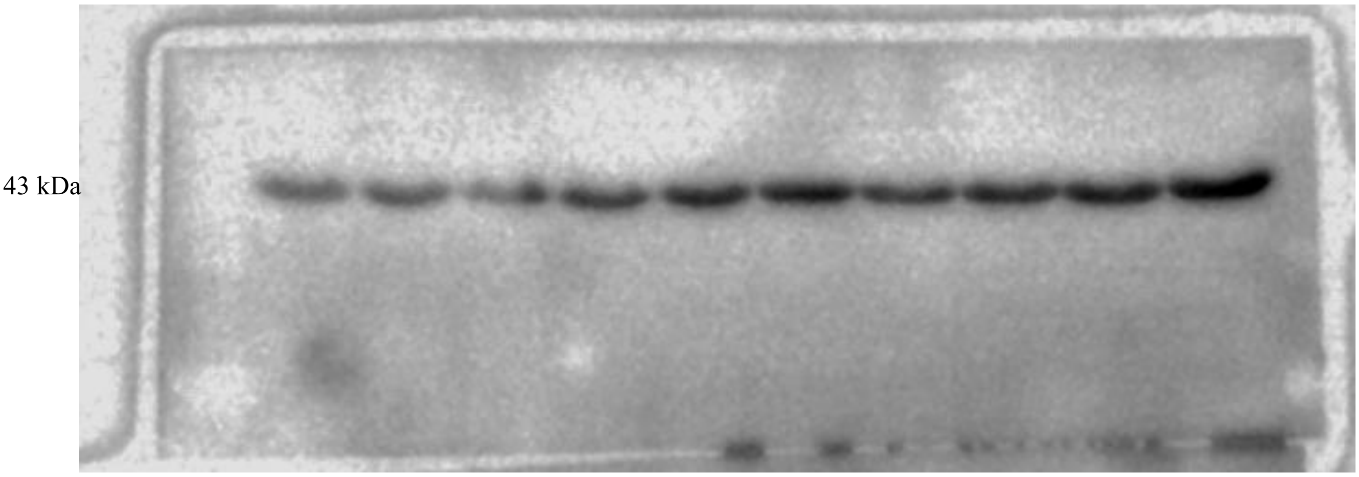

Lanes of Blot 3&4:

M: PageRuler Prestained Protein Ladder, 10 to 180 kDa (Thermo Scientific, #26619)

<u>**1-5 lanes:** whole cell lysates from AX2</u>

1: 0 min starvation (non-starved cells)

2: 10 min starvation

3: 20 min starvation

4: 30 min starvation

5: 60 min starvation

<u>**6-10 lanes:** whole cell lysates from *rheb*$^{-}$ (clone: rheb-_I_12_1)</u>

1: 0 min starvation (non-starved cells)

2: 10 min starvation

3: 20 min starvation

4: 30 min starvation

5: 60 min starvation

Figure 3E (i)

Blot 5: Western blotting: phospho-AMPK (T172) (rabbit, 1:1000)

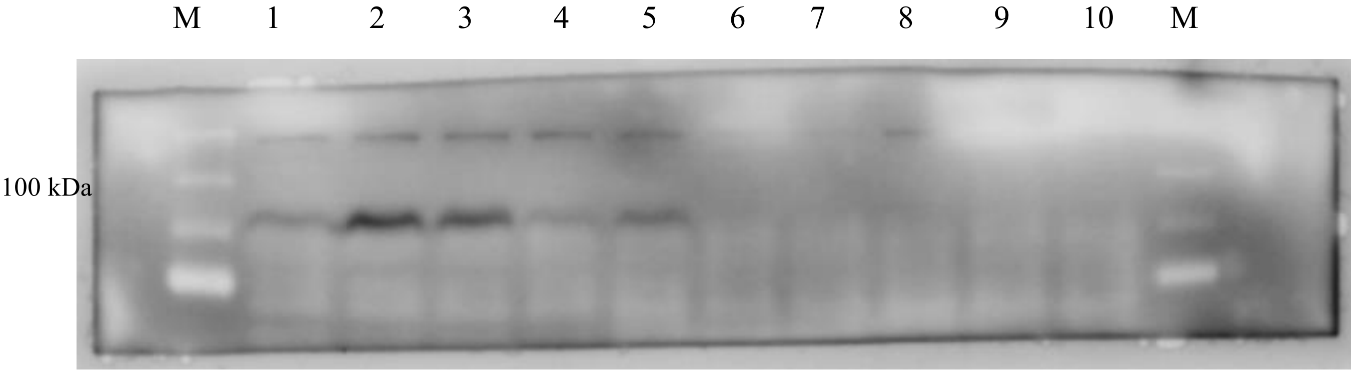

Blot 6: Western blotting: phospho-4E-BP1 (T37/46) (rabbit, 1:1000)

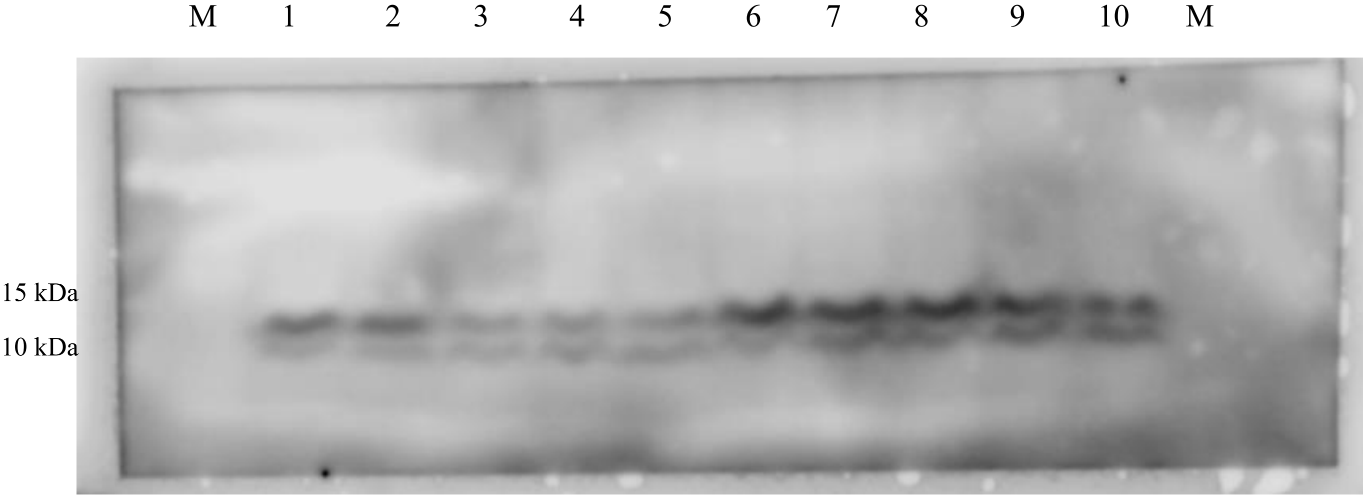

Blot 7: Western blotting: MCCC1 (Streptavidin, Alexa Fluo 680 conjugate, 1:1000)

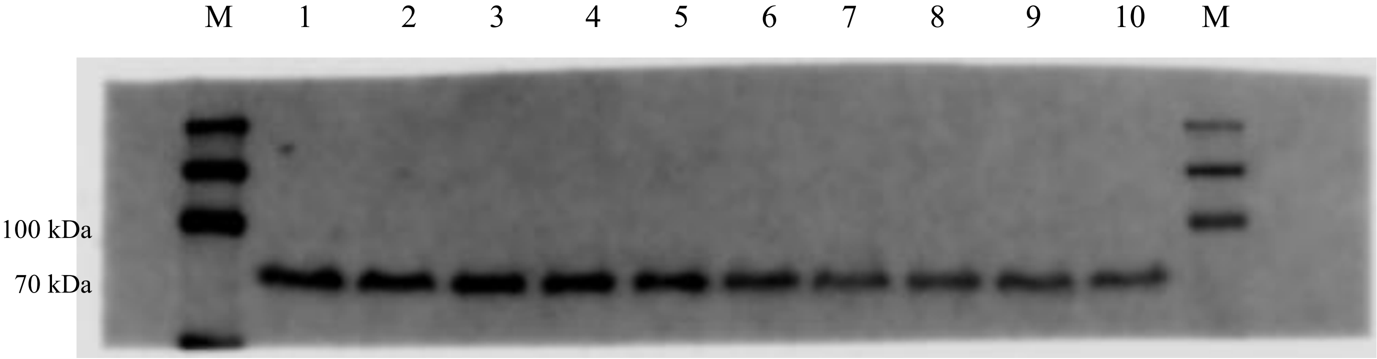

Lanes of Blot 5, 6&7:

M: PageRuler Prestained Protein Ladder, 10 to 180 kDa (Thermo Scientific, #26619)

<u>**1-5 lanes:** whole cell lysates from AX3</u>

1: 0 min starvation (non-starved cells)

2: 10 min starvation

3: 20 min starvation

4: 30 min starvation

5: 60 min starvation

<u>6-10 lanes:</u> <u>whole cell lysates from *ampk*$^-$</u>

1: 0 min starvation (non-starved cells)

2: 10 min starvation

3: 20 min starvation

4: 30 min starvation

5: 60 min starvation

Figure 4A (i)

Blot 8: Western blotting: phospho-4E-BP1 (T37/46) (rabbit, 1:1000)

M 1 2 3 4 5 6 7 8 9

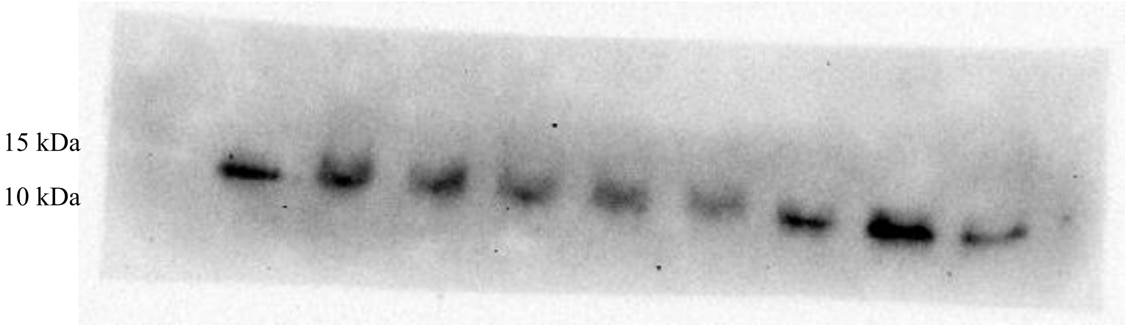

Blot 9: Western blotting: MCCC1 (Streptavidin, Alexa Fluo 680 conjugate, 1:1000)

M 1 2 3 4 5 6 7 8 9

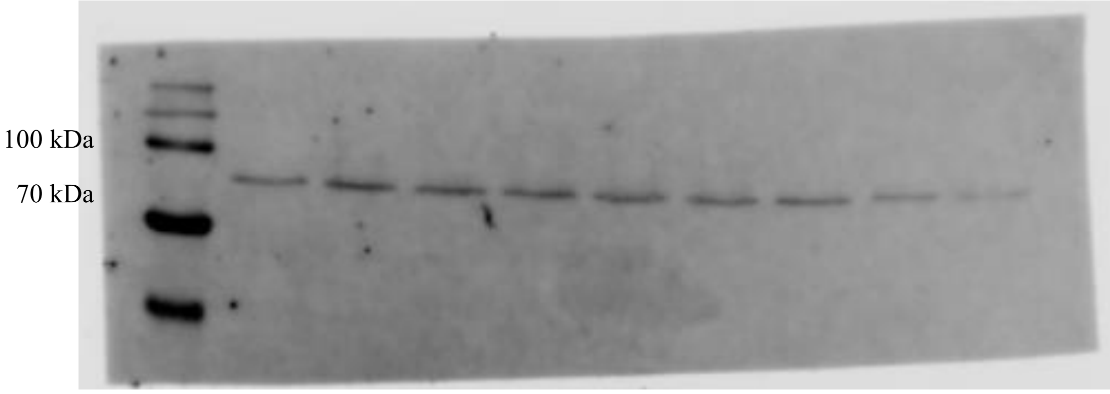

Lanes of Blot 8&9:

M: PageRuler Prestained Protein Ladder, 10 to 180 kDa (Thermo Scientific, #26619)

<u>1-3 lanes:</u> <u>whole cell lysates from growing AX2 with 500 nM rapamycin</u>

1: 0 min (untreated cells)

2: 5 min with 500 nM rapamycin

3: 10 min with 500 nM rapamycin

4: 15 min with 500 nM rapamycin

<u>5-7 lanes:</u> <u>whole cell lysates from growing AX2 with 500 nM AZD8055</u>

5: 5 min with 500 nM AZD8055

6: 10 min with 500 nM AZD8055

7: 15 min with 500 nM AZD8055

<u>**8-9 lanes:** control experiment</u>

8: 0 min starvation (non-starved and untreated with mTOR inhibitors)

9: 60 min starvation (untreated with mTOR inhibitors)

Figure 6B (i)

Blot 10: Western blotting: H3K4me3 (rabbit, 1:2000)

M 1 2 3 4 5 M 6 7 8 9 10

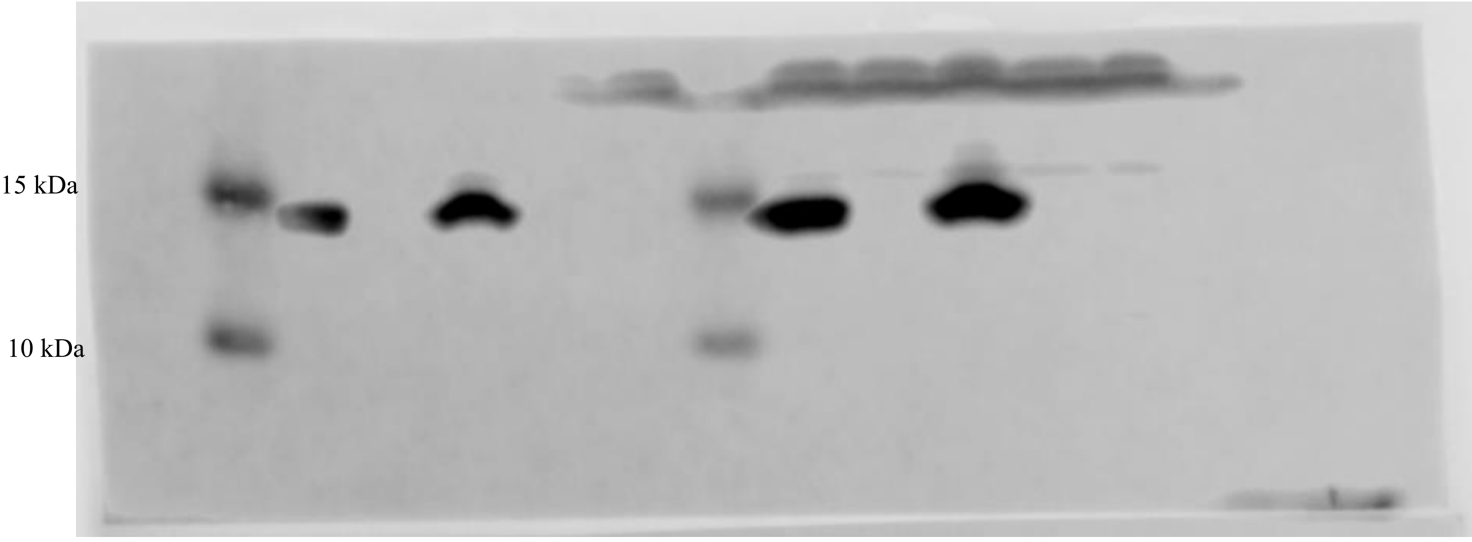

Blot 11: Western blotting: MCCC1 (Streptavidin, Alexa Fluo 680 conjugate, 1:1000)

M 1 2 3 4 5 M 6 7 8 9 10

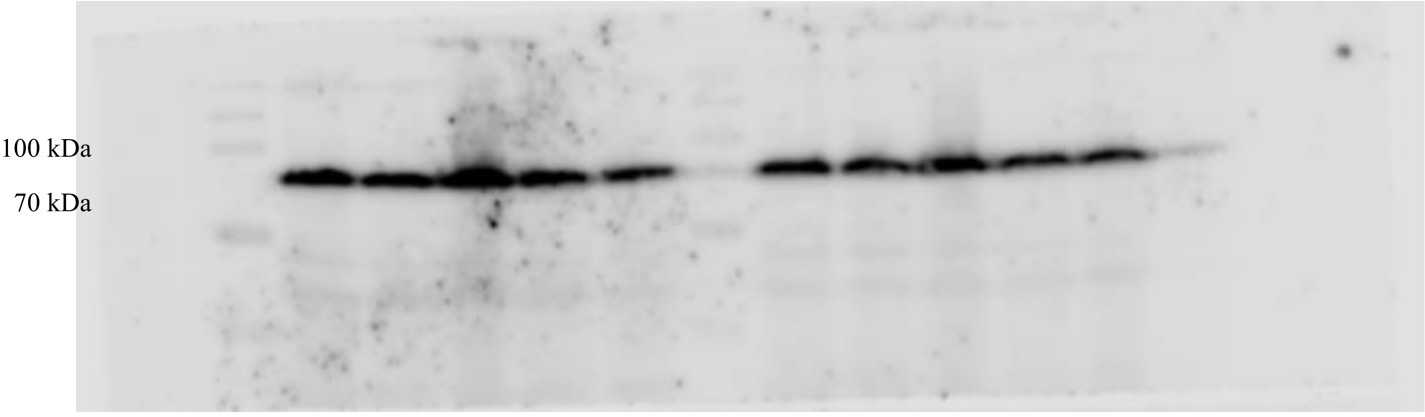

Lanes of Blot 10&11:

M: PageRuler Prestained Protein Ladder, 10 to 180 kDa (Thermo Scientific, #26619)

<u>**1-5 lanes:** repeat #1: whole cell lysates of AX2 and *set1*$^-$ and its rescue cell lines (N1425Q and C1474A)</u>

1: AX2

2: *set1*$^-$

3: GFP-Set1

4: GPF-Set1:N1425Q

5: GFP-Set1:C1474A

6-10 lanes: repeat #2: whole cell lysates of AX2 and *set1*$^-$ and its rescue cell lines (N1425Q and C1474A)

1: AX2

2: *set1*$^-$

3: GFP-Set1

4: GPF-Set1:N1425Q

5: GFP-Set1:C1474A

Figure 6D (i)

Blot 12: Western blotting: phospho-4E-BP1 (T37/46) (rabbit, 1:1000)

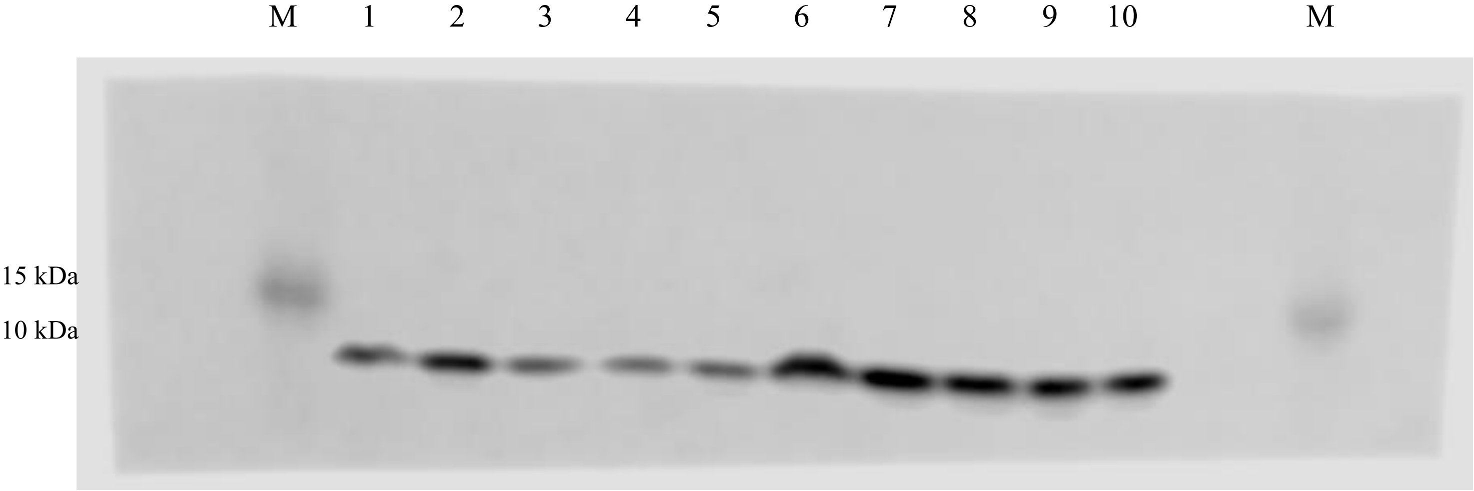

Blot 13: Western blotting: MCCC1 (Streptavidin, Alexa Fluo 680 conjugate, 1:1000)

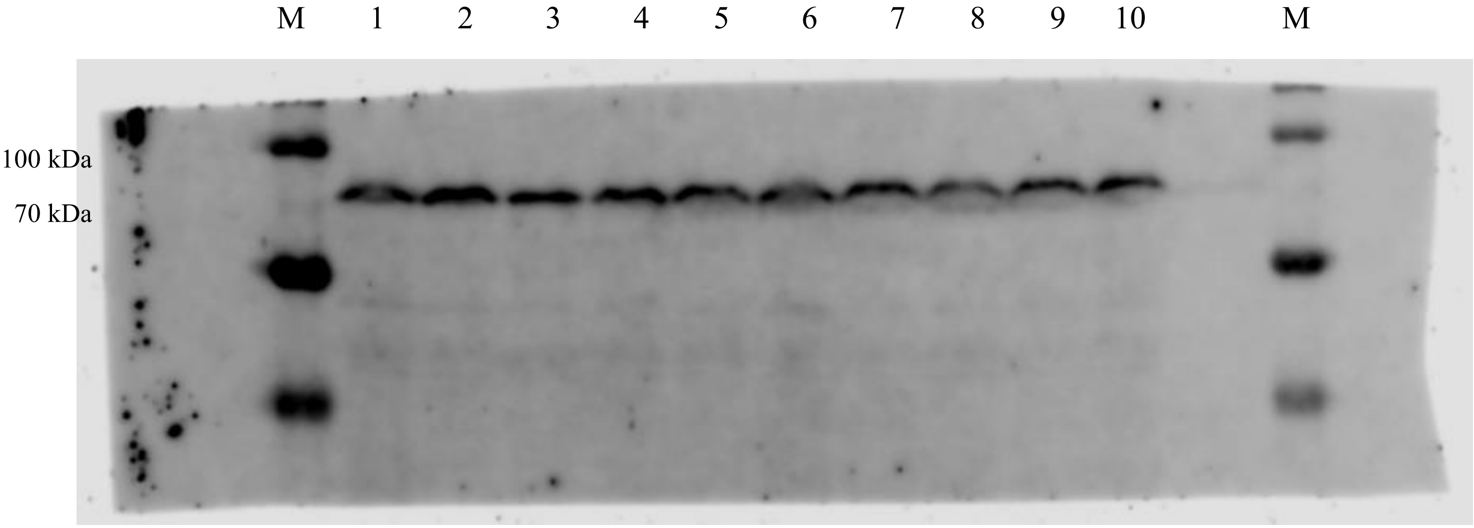

Lanes of Blot 12&13:

M: PageRuler Prestained Protein Ladder, 10 to 180 kDa (Thermo Scientific, #26619)

1-5 lanes: whole cell lysates from AX2

1: 0 min starvation (non-starved cells)

2: 10 min starvation

3: 20 min starvation

4: 30 min starvation

5: 60 min starvation

6-10 lanes: whole cell lysates from *set1*$^{-}$

1: 0 min starvation (non-starved cells)

2: 10 min starvation

3: 20 min starvation

4: 30 min starvation

5: 60 min starvation

Figure 1A

	R1(% green spores)	R2	R3	P value (student t test)	Significance
10LAX2_90UL*tpc2*$^-$	26.0	28.0	22.0	0.001	***
10L*tpc2*$^-$_90ULAX2	9.2	10.0	9.0		
50LAX2_50UL*tpc2*$^-$	72.0	60.0	61.0	0.012	*
50L*tpc2*$^-$_50ULAX2	49.0	45.0	47.0		
90LAX2_10UL*tpc2*$^-$	92.0	84.0	93.0	0.015	*
90L*tpc2*$^-$_10ULAX2	77.0	72.0	78.0		

Figure 1B

DIF-1 (nM)	R1	R2	R3	R1	R2	R3
	AX2+ecmB-GAL (% beta-gal activity)			tpc2$^-$+ecmB-GAL. (% beta-gal activity)		
0	0	0	0	0	0	0
10	3.457074	6.693065	11.43999	27.87978	45.85322	59.25166
20	7.674293	13.06033	29.15145	43.76006	67.12159	64.81286
40	21.88266	35.79114	54.07944	49.30687	85.07131	60.75858
50	23.8767	46.71943	60.27203	73.94334	78.64995	86.75649
60	49.40389	53.39606	84.35486	82.29023	88.36267	75.11087
80	62.40436	79.32705	100	100	76.87218	100
100	100	100	100	100	100	100

Two-way RM ANOVA	Matching: Across row
Alpha	0.05

Source of Variation	% of total variation	P value	P value summary	Significant?
Interaction	4.947	0.0213	*	Yes
DIF conc	76.97	<0.0001	****	Yes
AX2 vs tpc2-	9.68	<0.0001	****	Yes
Subjects (matching)	5.034	0.2155	ns	No

ANOVA table	SS	DF	MS	F (DFn, DFd)	P value
Interaction	2910	7	415.7	F (7, 16) = 3.355	P=0.0213
DIF conc	45278	7	6468	F (7, 16) = 34.95	P<0.0001
AX2 vs tpc2-	5695	1	5695	F (1, 16) = 45.96	P<0.0001
Subjects (matching)	2961	16	185.1	F (16, 16) = 1.494	P=0.2155
Residual	1983	16	123.9		

Figure 1C

Figure 1C (i)	R1	R2	R3	R1	R2	R3
Salt (mM)	AX2+ecmB-GAL_NaCl			AX2+ecmB-GAL_Imidazole		
0	100	100	100	100	100	100
10	88.19	81.18	128.97	41.43	89.92	11.85
20	135.5	37.52	33.7	5.51	8.93	4.27
30	75.57	44.18	11.59	5.51	19.73	3.77
40	25.69	9.86	8.82	5.63	14.32	3.86

Figure 1C (ii)	R1	R2	R3	R1	R2	R3
Salt (mM)	*tpc2*$^-$+ecmB-GAL_NaCl			*tpc2*$^-$+ecmB-GAL_Imidazole		
0	100	100	100	100	100	100
10	80	48.33	86.62	34.12	19.26	42.95
20	56.94	21.17	9.56	5.25	5.82	2.35
30	23.11	26.39	3.02	1.46	12.51	1.83
40	8.52	20.19	2.3	1.53	5.12	1.73

Fig 1C(i) Two-way RM ANOVA	Matching: Across row	
Alpha		0.05

Source of Variation	% of total variation	P value	P value summary	Significant?
Interaction	7.893	0.332	ns	No
conc of imidazole/NaCl	55.47	0.000	***	Yes
AX2 +NaCl or AX2 + imi	12.76	0.016	*	Yes
Subjects (matching)	8.781	0.797	ns	No

ANOVA table		DF	MS	F (DFn, DFd)	P value
Interaction	4479	4	1120	F (4, 10) = 1.307	P=0.3319
conc of imidazole/NaCl	1472	4	7868	F (4, 10) = 15.79	P=0.0003
AX2 +NaCl or imidazole	7240	1	7240	F (1, 10) = 8.449	P=0.0157
Subjects (matching)	4983	10	498.3	F (10, 10) = 0.5815	P=0.7971
Residual	8569	10	856.9		

Fig 1c(ii). Two-way RM ANOVA

ANOVA	Matching: Across row			
Alpha	0.05			

Source of Variation	% of total variation	P value	P value summary	Significant?
Interaction	3.336	0.0242	*	Yes
salt conc	85.09	<0.0001	****	Yes
tpc2- + NaCl or tpc2- + imi	4.867	0.0004	***	Yes
Subjects (matching)	4.864	0.0711	ns	No

ANOVA table	SS	DF	MS	F (DFn, DFd)	P value
Interaction	1454	4	363.4	F (4, 10) = 4.521	P=0.0242
salt conc	37070	4	9267	F (4, 10) = 43.73	P<0.0001
tpc2- + NaCl or tpc2- + imi	2120	1	2120	F (1, 10) = 26.38	P=0.0004
Subjects (matching)	2119	10	211.9	F (10, 10) = 2.637	P=0.0711
Residual	803.8	10	80.38		

Figure 1D

	R1 (%green spores)	R2(%green spores)	R3(%green spores)	P value (student t test)	Significance
10LNaCl_90ULImi	5.43	10.07	6.56	0.041	*
10LImi_90ULNaCl	10.94	17.64	19.1		
50LNaCl_50ULImi	27.34	42.89	49.42	0.054	ns
50LImi_50ULNaCl	51.83	69.78	70.08		
90LNaCl_10ULImi	88.32	89.31	83.46	0.113	ns
90LImi_10ULNaCl	89.85	91.12	91.51		

Figure 1E

	R1 (%green spores)	R2 (%green spores)	R3 (%green spores)	P value (student t test)	Significance
10LAX2NaCl_90UL*tpc2*$^{-}$Imi	10.27	9.96	10.09	0.393	ns
10L*tpc2*$^{-}$Imi_90ULAX2NaCl	10.14	10.27	10.19		
50LAX2NaCl_50UL*tpc2*$^{-}$Imi	50.85	50.21	50.59	0.535	ns
50L*tpc2*$^{-}$Imi_50ULAX2NaCl	50.31	50.7	50.13		
90LAX2NaCl_10UL*tpc2*$^{-}$Imi	89.98	89.73	89.61	0.882	ns
90L*tpc2*$^{-}$Imi_10ULAX2NaCl	90.21	89.69	89.53		

Figure 2A

(ii)	R1	R2	R3	R1	R2	R3		
Time (min)		20 mM NaCl			20 mM imidazole		P value (student t test)	Significance
0	100	100	100	73.5	12.3	59.4	0.049377533	*
10	179.6	114.6	87.7	143.5	35.2	80.5		
20	143	100.4	79.7	90.3	12.1	29.3		
30	95.4	80.6	73.7	18.6	2.4	23.4		

Two-way RM ANOVA	Matching: Stacked
Alpha	0.05

Source of Variation	% of total variation	P value	P value summary	Significant?
Interaction	1.413	0.7971	ns	No
timepoint	29.96	0.0006	***	Yes
NaCl vs imidazole	36.63	0.0474	*	Yes
Subjects (matching)	18.3	0.0063	**	Yes

ANOVA table	SS	DF	MS	F (DFn, DFd)	P value
Interaction	842.3	4	210.6	F (4, 16) = 0.4125	P=0.7971
timepoint	17863	4	4466	F (4, 16) = 8.748	P=0.0006
NaCl vs imidazole	21838	1	21838	F (1, 4) = 8.006	P=0.0474
Subjects (matching)	10910	4	2728	F (4, 16) = 5.343	P=0.0063
Residual	8168	16	510.5		

Figure 2C

	Rapamycin (nM)	R1	R2	R3	R1	R2	R3	P value (student t test)
			AX2			*tpc2*$^{-}$		
com HL5	0	0	0	0	0	0	0	-
0	0	0	0	0	0	0	0	-
5	5	1	0	1	0	1	1	1.000
10	10	2	1	1	1	1	1	0.374
50	50	3	2	2	2	2	1	0.230
100	100	3	3	3	2	2	1	0.016
500	500	3	4	4	3	3	4	0.519
KK2	0	5	5	5	5	5	5	-

Two-way RM ANOVA — Matching: Stacked

Alpha — 0.05

Source of Variation	% of total variation	P value	P value summary	Significant?
Interaction	1.683	0.1135	ns	No
rapamycin conc	93.51	<0.0001	****	Yes
AX2 or tpc2-	0.9615	0.0161	*	Yes
Subjects (matching)	0.2404	0.7596	ns	No

ANOVA table	SS	DF	MS	F (DFn, DFd)	P value
Interaction	2.333	7	0.3333	F (7, 28) = 1.867	P=0.1135
rapamycin conc	129.7	7	18.52	F (7, 28) = 103.7	P<0.0001
AX2 or tpc2-	1.333	1	1.333	F (1, 4) = 16	P=0.0161
Subjects (matching)	0.3333	4	0.08333	F (4, 28) = 0.4667	P=0.7596
Residual	5	28	0.1786		

Figure 2D

	Rapamycin (nM)	R1	R2	R3	R1	R2	R3	P value (student t test)
		20 mM NaCl			20 mM imidazole			
com HL5	0	1	0	0	0	0	0	0.374
0	0	0	0	0	0	0	0	-
5	5	1	1	1	2	3	2	0.016
10	10	1	2	0	2	2	2	0.158
20	20	1	2	2	2	4	2	0.251
50	50	3	3	2	3	2	3	1.000
100	100	3	4	2	3	4	3	0.643
KK2	0	5	5	5	5	5	2	0.374

Two-way RM ANOVA	Matching: Stacked
Alpha	0.05

Source of Variation	% of total variation	P value	P value summary	Significant?
Interaction	5.446	0.088	ns	No
rapamycin conc	79.69	<0.0001	****	Yes
NaCl or imidazole	0.858	0.3603	ns	No
Subjects (matching)	3.222	0.1087	ns	No

ANOVA table	SS	DF	MS	F (DFn, DFd)	P value
Interaction	6.479	7	0.9256	F (7, 28) = 2.019	P=0.0880
rapamycin conc	94.81	7	13.54	F (7, 28) = 29.55	P<0.0001
NaCl or imidazole	1.021	1	1.021	F (1, 4) = 1.065	P=0.3603
Subjects (matching)	3.833	4	0.9583	F (4, 28) = 2.091	P=0.1087
Residual	12.83	28	0.4583		

Figure 3A

Figure 3A (ii)	R1	R2	R1	R2		
Time (min)	AX2		*rheb*$^-$		P value (student t test)	Significance
0	100	100	20.2	9.5	0.003924423	**
10	59.7	44.3	23.8	10.8		
20	64.3	57.4	24	13.8		
30	46.9	47.2	24.2	20.2		
60	48.8	52.6	7.3	22.6		

Two-way RM ANOVA	Matching: Stacked
Alpha	0.05

Source of Variation	% of total variation	P value	P value summary	Significant?
Interaction	15.59	0.0021	**	Yes
time	11.6	0.0054	**	Yes
AX2 vs rheb-_I_12_1	69.52	0.0042	**	Yes
Subjects (matching)	0.5918	0.4527	ns	No

ANOVA table	SS	DF	MS	F (DFn, DFd)	P value
Interaction	2218	4	554.4	F (4, 8) = 11.54	P=0.0021
time	1650	4	412.6	F (4, 8) = 8.589	P=0.0054
AX2 vs rheb-_I_12_1	9892	1	9892	F (1, 2) = 235	P=0.0042
Subjects (matching)	84.2	2	42.1	F (2, 8) = 0.8765	P=0.4527
Residual	384.3	8	48.03		

Figure 3B

		R1	R2	R3	R1	R2	R3	
	[Rapamycin] (nM)		AX2			*rheb*$^-$ (I_12_1)		P value (student t test)
com HL5	0	0	0	0	0	0	0	-
0	0	0	0	0	0	0	0	-
10	10	0	1	0	1	2	2	0.047
50	50	1	1	1	2	3	4	0.026
100	100	2	3	2	4	3	1	0.742
500	500	4	3	3	4	4	4	0.116
KK2	0	5	5	5	5	5	5	-

Two-way RM ANOVA Matching: Stacked

Alpha 0.05

Source of Variation	% of total variation	P value	P value summary	Significant?
Interaction	3.777	0.0547	ns	No
rapamycin conc	87.01	<0.0001	****	Yes
AX2 or rheb- (l_12_1 clone)	2.775	0.0029	**	Yes
Subjects (matching)	0.2628	0.9035	ns	No

ANOVA table	SS	DF	MS	F (DFn, DFd)	P value
Interaction	5.476	6	0.9127	F (6, 24) = 2.447	P=0.0547
rapamycin conc	126.1	6	21.02	F (6, 24) = 56.36	P<0.0001
AX2 or rheb- (l_12_1 clone)	4.024	1	4.024	F (1, 4) = 42.25	P=0.0029
Subjects (matching)	0.381	4	0.09524	F (4, 24) = 0.2553	P=0.9035
Residual	8.952	24	0.373		

Figure 3C

	R1	R2	R3	P value (student t test)	Significance
10LAX2_90UL*rheb*$^-$	8.53	9.59	9.55	0.001	**
10L*rheb*$^-$_90ULAX2	12.14	12.38	12.65		
50LAX2_50UL*rheb*$^-$	45.13	44.76	47.67	0.006	**
50L*rheb*$^-$_50ULAX2	50.74	51.69	53.04		
90LAX2_10UL*rheb*$^-$	87.29	86.77	84.13	0.015	*
90L*rheb*$^-$_10ULAX2	92.77	94.19	90.09		

Figure 3D

	R1	R2	R3	R4	R1	R2	R3	R4
DIF-1 (nM)	AX2+ecmB-GAL				*rheb*$^-$+ecmB-GAL			
0	0	0	0	0	0	0	0	0
10	14.29184	50	28.34601	73.41739	32.25588	40.38306	-48.5729	23.53305
20	71.43775	83.325	47.24335	74.05157	64.51176	23.07604	-42.8559	52.94273
50	71.43775	100	125.1949	92.40497	93.54495	49.99423	2.854204	47.05727
100	100	100	100	100	100	100	100	100

Two-way RM ANOVA	Matching: Across row			
Alpha	0.05			

Source of Variation	% of total variation	P value	P value summary	Significant?
Interaction	5.609	0.2101	ns	No
DIF conc	61.2	<0.0001	****	Yes
AX2 vs rheb-	7.639	0.0088	**	Yes
Subjects (matching)	12.92	0.4829	ns	No

ANOVA table	SS	DF	MS	F (DFn, DFd)	P value
Interaction	4451	4	1113	F (4, 15) = 1.665	P=0.2101
DIF conc	48567	4	12142	F (4, 15) = 17.76	P<0.0001
AX2 vs rheb-	6062	1	6062	F (1, 15) = 9.071	P=0.0088
Subjects (matching)	10252	15	683.5	F (15, 15) = 1.023	P=0.4829
Residual	10024	15	668.3		

Figure 3E

Figure 3E (ii)

Time (min)	R1	R2	R3	R1	R2	R3	P value (student t test)	Significance
	AX3_p-4E-BP1(T37/46)			ampk-_p-4E-BP1(T37/46)				
0	100	100	100	135.9	179.4	178.7	0.01086	*
10	73.5	82	80.9	89.7	57.8	201.3		
20	56.6	19.8	27.2	72.1	34.7	196.1		
30	31.8	13.1	38.9	52.6	37.6	147.7		
60	36.2	9.5	41.8	51.5	28	85		

Two-way RM ANOVA	Matching: Stacked
Alpha	0.05

Source of Variation	% of total variation	P value	P value summary	Significant?
Interaction	2.07	0.6056	ns	No
Timepoint	35.79	0.0001	***	Yes
AX3 vs ampk- (p-4ebp1)	20.26	0.1755	ns	No
Subjects (matching)	29.98	0.0003	***	Yes

ANOVA table	SS	DF	MS	F (DFn, DFd)	P value
Interaction	1849	4	462.1	F (4, 16) = 0.6961	P=0.6056
Timepoint	31963	4	7991	F (4, 16) = 12.04	P=0.0001
AX3 vs AMPK- (p-4ebp1)	18096	1	18096	F (1, 4) = 2.704	P=0.1755
Subjects (matching)	26772	4	6693	F (4, 16) = 10.08	P=0.0003
Residual	10623	16	663.9		

Figure 3F

	R1	R2	R3	P value (student t test)	Significance
10LAX3_90UL*ampk*$^-$	11.71	19.33	18.51	0.039	*
10L*ampk*$^-$_90ULAX3	7.91	9.82	9.36		
50LAX3_50UL*ampk*$^-$	56.87	64.09	54.4	0.008	**
50L*ampk*$^-$_50ULAX3	39.2	40.59	45.39		
90LAX3_10UL*ampk*$^-$	91.01	91.07	92.75	0.007	**
90L*ampk*$^-$_10ULAX3	74.71	83.16	77.14		

Figure 3G

DIF-1 (nM)	R1	R2	R3	R1	R2	R3
	AX3+ecmB-GAL			*ampk*$^{-}$+ecmB-GAL		
0	0	0	0	0	0	0
10	67.74194	56.75127	123.8095	70.42921	101.709	79.3545
20	79.56774	78.16456	121.4286	89.84194	95.72564	95.87095
50	94.62258	94.62025	113.4905	93.45415	92.73462	144.6698
100	100	100	100	100	100	100

Two-way RM ANOVA	Matching: Across row
Alpha	0.05

Source of Variation	% of total variation	P value	P value summary	Significant?
Interaction	0.1862	0.9856	ns	No
DIF-1 conc	87.53	<0.0001	****	Yes
AX3 vs ampk-	0.07235	0.7258	ns	No
Subjects (matching)	6.654	0.3908	ns	No

ANOVA table	SS	DF	MS	F (DFn, DFd)	P value
Interaction	96.78	4	24.2	F (4, 10) = 0.08372	P=0.9856
DIF-1 conc	45505	4	11376	F (4, 10) = 32.88	P<0.0001
AX3 vs ampk-	37.62	1	37.62	F (1, 10) = 0.1302	P=0.7258
Subjects (matching)	3460	10	346	F (10, 10) = 1.197	P=0.3908
Residual	2890	10	289		

Figure 4A

Figure 4A (ii)		R1	R2	R3		
Time (min)			500 nM Rapa		P value (student t test)	Significance
	0	100	100	100		
	5	73	60	59	0.0013	**
	10	40	65	58	0.0036	**
	15	19	40	20	0.0004	***

Figure 4A (iii)

Starvation time (min)	R1	R2	R3	P value (student t test)	Significance
0	100	100	100		
60	74	46	33	0.0155	*

Figure 4B

	R1	R2	R3		
		AX2		P value (student t test)	Significance
10LR+_90ULR-	21.75	18.66	20.33	0.0005	***
10LR-_90ULR+	9.13	7.34	9.45		
90LR+_10ULR-	96.38	96.11	92.64	0.0078	**
90LR-_10ULR+	81	85.25	76.2		

Figure 4C

	R1	R2		
Rapamycin (nM)	100 nM DIF-1_45 min		P value (student t test)	Significance
0	100	100	0.4030	ns
10	117.97	100.46	0.402951482	ns
100	209.05	207.83	0.00003	****
500	249.76	216.57	0.015177443	*

	R1	R2		
Rapamycin (nM)	50 nM DIF-1_45 min		P value (student t test)	Significance
0	100	100	0.3807	ns
10	100.09	101.65	0.380731938	ns
100	158.83	123.62	0.14400	ns
500	161.47	154.25	0.003870175	**

Figure 4D

	R1	R2	R3	R4	P value (student t test)	Significance
		AX2+ecmB-GAL				
0	100	100	100	100	-	
10	39.21	90.81	79.68	66.03	0.031501	*
20	65.71	107.92	71.76	55.46	0.07281	ns
50	58.57	87.74	51.27	52.97	0.00462	**
100	10.39	31.44	23.58	53.09	0.000223	***

Figure 5B

	R1	R2	R3	R1	R2	R3
		rrgA			rigA	
AX2	1	1	1	1	1	1
tpc2⁻	10.66484	3.476142	4.082366	0.171266	0.999103	1.554801

Figure 5C

	R1	R2	R3	R1	R2	R3
		rrgA			rigA	
DMSO	1	1	1	1	1	1
100nM Rapa	0.478711	2.506468	5.148857	4.004644	8.333613	2.326284

Figure 5B

	R1	R2	R3	R1	R2	R3
		rrgA/rigA			log10(rrgA/rigA)	
AX2	1	1	1	0	0	0
tpc2⁻	62.27046	3.479261	2.625651	1.794282	0.541487	0.419237

Figure 5C

	R1	R2	R3	R1	R2	R3
		rrgA/rigA			log10(rrgA/rigA)	
DMSO	1	1	1	0	0	0
100nM Rapa	0.119539	0.300766	2.21334	-0.92249	-0.52177	0.345048

Figure 6A

Figure 6A (i)	R1	R2	R3	R1	R2	R3
		rrgA			rigA	
AX2	1	1	1	1	1	1
set1$^-$	5.498708	1.432001	2.817031	0.812641	0.762563	0.309196

Figure 6A (i)	R1	R2	R3	R1	R2	R3
		rrgA/rigA			log10(rrgA/rigA)	
AX2	1	1	1	0	0	0
set1$^-$	6.766469	1.877877	9.110831	0.830362	0.273667	0.959558

Figure 6A (ii)	R1	R2	R3	R4	P value (student t test)	Significance
10LAX2_90UL*set1*$^-$	13.51551	18.14059	14.49275	17.2956	0.0002	***
10L*set1*$^-$_90ULAX2	6.923751	7.26257	5.084746	6.666667		
50LAX2_50UL*set1*$^-$	51.26183	61.16071	64.22018	70.83333	0.0010	**
50L*set1*$^-$_50ULAX2	35.6564	32.36246	38.7218	38.20225		
90LAX2_10UL*set1*$^-$	94.75113	92.55319	95.58824	92.02899	0.0257	*
90L*set1*$^-$_10ULAX2	78.48411	90	89.38547	83.4728		

Figure 6A (iii)	R1	R2	R3	R4	R1	R2	R3	R4
DIF-1 (nM)		AX2+ecmB-GAL				*set1*$^-$+ecmB-GAL		
0	0	0	0	0	0	0	0	0
10	-6.12903	-30.3148	12.44972	13.09234	3.779539	76.99047	37.4773	37.29256
20	-11.8281	-40.7506	69.57338	31.41705	94.96093	81.41609	77.2973	66.70126
50	50.32258	-0.29056	107.2981	100.539	75.59079	104.4243	104.5946	89.10789
100	100	100	100	100	100	100	100	100

Two-way RM ANOVA | Matching: Across row
Alpha 0.05

Source of Variation	% of total variation	P value	P value summary	Significant?
Interaction	7.552	0.1251	ns	No
DIF-1 conc	61.83	<0.0001	****	Yes
AX2 vs set1-	8.649	0.0068	**	Yes
Subjects (matching)	8.776	0.7805	ns	No

ANOVA table	SS	DF	MS	F (DFn, DFd)	P value
Interaction	6706	4	1677	F (4, 15) = 2.147	P=0.1251
DIF-1 conc	54901	4	13725	F (4, 15) = 26.42	P<0.0001
AX2 vs set1-	7680	1	7680	F (1, 15) = 9.835	P=0.0068
Subjects (matching)	7792	15	519.5	F (15, 15) = 0.6652	P=0.7805
Residual	11714	15	780.9		

Figure 6B

Figure 6B (i)

	R1	R2	R3	R1	R2	R3
		rrgA			rigA	
AX2	1	1	1	1	1	1
H3bK4A	2.000344	4.33644	1.627479	0.826451	0.715045	0.242677

	R1	R2	R3	R1	R2	R3
		rrgA/rigA			log10(rrgA/rigA)	
AX2	1	1	1	0	0	0
H3bK4A	2.420402	6.064567	6.706354	0.383888	0.7828	0.826486

Figure 6B (ii)

	R1	R2	R3	R4	P value (student t test)	Significance
10LAX2_90ULH3bK4A	14.35	11.71	11.23	19.27	0.0256	*
10LH3bK4A_90ULAX2	7.48	8.55	8.86	9.41		
50LAX2_50ULH3bK4A	53.81	71.21	56	57.68	0.0098	**
50LH3bK4A_50ULAX2	31.29	46.55	42.59	42.15		
90LAX2_10ULH3bK4A	90.5	91.67	92.86	90.91	0.0398	*
90LH3bK4A_10ULAX2	85.95	87.78	76.12	68.29		

Figure 6C

Figure 6C (ii)

	GFP-Set1 labelled_AX2 unlabelled			P value (student t test)	Significance
10LGFP-Set1+90ULAX2	10.48	10.1	9.89	0.000727007	***
10L*set1*$^-$+90ULAX2	6.923751	7.61	6.666667		
50LGFP-Set1+50ULAX2	50.67	50.57	50.2	0.063679667	ns
50L*set1*$^-$+50ULAX2	35.6564	48.24	38.20225		
90LGFP-Set1+10ULAX2	89.72	90.39	89.55	0.012011812	*
90L*set1*$^-$+10ULAX2	78.48411	74.87	83.4728		

Figure 6D

Time (min)	R1	R2	R3	R4	R1	R2	R3	R4	P value (student t test)
			AX2				*set1*$^-$		
0	100	100	100	100	97.4	677.3	260.7	103.3	0.22402
10	98.3	77.2	65.7	94.1	133.1	469.2	332.1	129.8	
20	95.6	65	32.5	67.9	107	408.9	64.5	126.5	
30	67.8	66.8	51.4	78.2	116.7	202.4	58.2	108	
60	56.4	47.3	2.5	74.2	95.8	195.1	69.5	100.2	

Two-way RM ANOVA	Matching: Stacked
Alpha	0.05

Source of Variation	% of total variation	P value	P value summary	Significant?
Interaction	4.504	0.2849	ns	No
timepoint	11.88	0.0212	*	Yes
AX2 vs set1-	22.17	0.1227	ns	No
Subjects (matching)	41.25	<0.0001	****	Yes

ANOVA table	SS	DF	MS	F (DFn, DFd)	P value
Interaction	29618	4	7405	F (4, 24) = 1.337	P=0.2849
timepoint	78108	4	19527	F (4, 24) = 3.527	P=0.0212
AX2 vs set1-	145781	1	145781	F (1, 6) = 3.224	P=0.1227
Subjects (matching)	271265	6	45211	F (6, 24) = 8.166	P<0.0001
Residual	132868	24	5536		

Figure 6E

		R1	R2	R3	R1	R2	R3	
	[Rapamycin] (nM)		AX2			*set1*$^-$		P value (student t tes
com HL5	0	0	0	0	0	0	0	
0	0	0	0	0	0	0	0	
5	5	2	1	1	2	1	0	0.643
10	10	2	2	1	2	0	1	0.374
50	50	2	2	2	1	0	0	0.007
100	100	2	2	2	0	0	1	0.0075
500	500	3	3	4	0	0	0	0.0006
KK2	0	5	5	5	5	5	4	0.374

Two-way RM ANOVA	Matching: Stacked
Alpha	0.05

Source of Variation	% of total variation	P value	P value summary	Significant?
Interaction	10.8	<0.0001	****	Yes
Rapa	73.78	<0.0001	****	Yes
AX2 vs set1-	9.254	0.0043	**	Yes
Subjects (matching)	1.093	0.2272	ns	No

ANOVA table	SS	DF	MS	F (DFn, DFd)	P value
Interaction	14	7	2	F (7, 28) = 8.506	P<0.0001
Rapa	95.67	7	13.67	F (7, 28) = 58.13	P<0.0001
AX2 vs set1-	12	1	12	F (1, 4) = 33.88	P=0.0043
Subjects (matching)	1.417	4	0.3542	F (4, 28) = 1.506	P=0.2272
Residual	6.583	28	0.2351		

Second clone of *tpc2*$^-$ cells, monolayer assay

N = 1 (clone: TPC1E14)

DIF-1 (nM)	AX2+*ecmB*-GAL	tpc2+*ecmB*-GAL
0	0	0
10	3.457173471	39.59884202
20	7.674381554	62.15419079
40	21.88262417	70.0326027
50	23.87673882	105.0248139
60	49.40381173	116.8803419
80	62.4043709	142.034257
100	100	100

Second *rheb*$^-$ clone

mixing experiment (2 rep)

rheb-_I_3_1	R1	R2	P value (student t test)	Significance
10LAX2_90UL*rheb*$^-$	5.83	7.44	0.070555	ns
10L*rheb*$^-$_90ULAX2	17.72	26.16		
50LAX2_50UL*rheb*$^-$	35.39	32.42	0.008354	**
50L*rheb*$^-$_50ULAX2	50.93	52.26		
90LAX2_10UL*rheb*$^-$	75.09	73.91	0.010942	*
90L*rheb*$^-$_10ULAX2	90.66	94.26		

rapamycin aggregation assay (1 rep)

	Rapamycin (nM)	AX2	*rheb*$^-$_I_3_1
com HL5	0	0	0
0	0	0	0
10	10	1	2
50	50	1	4
100	100	1	4
500	500	3	4
KK2	0	5	5
